# Quantifying the legacy of the Chinese Neolithic on the maternal genetic heritage of Taiwan and Island Southeast Asia

**DOI:** 10.1007/s00439-016-1640-3

**Published:** 2016-02-13

**Authors:** Andreia Brandão, Ken Khong Eng, Teresa Rito, Bruno Cavadas, David Bulbeck, Francesca Gandini, Maria Pala, Maru Mormina, Bob Hudson, Joyce White, Tsang-Ming Ko, Mokhtar Saidin, Zainuddin Zafarina, Stephen Oppenheimer, Martin B. Richards, Luísa Pereira, Pedro Soares

**Affiliations:** 1 IPATIMUP (Institute of Molecular Pathology and Immunology of the University of Porto), Rua Dr. Roberto Frias s/n, 4200-465 Porto, Portugal; 2 i3S (Instituto de Investigação e Inovação em Saúde, Universidade do Porto), 4200 Porto, Portugal; 3 Department of Biological Sciences, School of Applied Sciences, University of Huddersfield Queensgate, Huddersfield, HD1 3DH UK; 4 ICBAS (Instituto Ciências Biomédicas Abel Salazar), Universidade do Porto, Rua de Jorge Viterbo Ferreira n.º 228, 4050-313 Porto, Portugal; 5 Faculty of Biological Sciences, University of Leeds, Leeds, LS2 9JT UK; 6 Centre for Global Archaeological Research, Universiti Sains Malaysia, 11800 Penang, Malaysia; 7 Life and Health Sciences Research Institute (ICVS), School of Health Sciences, University of Minho, Braga, Portugal; 8 ICVS/3B’s-PT Government Associate Laboratory, Braga/Guimarães, Portugal; 9 Department of Archaeology and Natural History, College of Asia and the Pacific, The Australian National University, Acton ACT, Canberra, 2601 Australia; 10 Department of Applied Social Studies, University of Winchester, Sparkford Road, Winchester, SO22 4NR UK; 11 Archaeology Department, University of Sydney, New South Wales, 2006 Australia; 12 Department of Anthropology, University of Pennsylvania Museum, 3260 South St., Philadelphia, USA; 13 Department of Obstetrics and Gynecology, National Taiwan University, Roosevelt Rd., Taipei, 10617 Taiwan; 14 Malaysian Institute of Pharmaceuticals and Nutraceuticals Malaysia, National Institutes of Biotechnology Malaysia, Penang, Malaysia; 15 Human Identification Unit, School of Health Sciences, Health Campus, Universiti Sains Malaysia, Kelantan, Malaysia; 16 School of Anthropology, Institute of Human Sciences, The Pauling Centre, University of Oxford, 58a Banbury Road, Oxford, OX2 6QS UK; 17 Faculty of Medicine, University of Porto, Al. Prof. Hernâni Monteiro, 4200-319 Porto, Portugal; 18 Department of Biology, CBMA (Centre of Molecular and Environmental Biology), University of Minho, Campus de Gualtar, 4710-057 Braga, Portugal

**Keywords:** Last Glacial Maximum, Sunda Shelf, Austronesian Language, Postglacial Expansion, Early Holocene Period

## Abstract

There has been a long-standing debate concerning the extent to which the spread of Neolithic ceramics and Malay-Polynesian languages in Island Southeast Asia (ISEA) were coupled to an agriculturally driven demic dispersal out of Taiwan 4000 years ago (4 ka). We previously addressed this question using founder analysis of mitochondrial DNA (mtDNA) control-region sequences to identify major lineage clusters most likely to have dispersed from Taiwan into ISEA, proposing that the dispersal had a relatively minor impact on the extant genetic structure of ISEA, and that the role of agriculture in the expansion of the Austronesian languages was therefore likely to have been correspondingly minor. Here we test these conclusions by sequencing whole mtDNAs from across Taiwan and ISEA, using their higher chronological precision to resolve the overall proportion that participated in the “out-of-Taiwan” mid-Holocene dispersal as opposed to earlier, postglacial expansions in the Early Holocene. We show that, in total, about 20 % of mtDNA lineages in the modern ISEA pool result from the “out-of-Taiwan” dispersal, with most of the remainder signifying earlier processes, mainly due to sea-level rises after the Last Glacial Maximum. Notably, we show that every one of these founder clusters previously entered Taiwan from China, 6–7 ka, where rice-farming originated, and remained distinct from the indigenous Taiwanese population until after the subsequent dispersal into ISEA.

**Electronic supplementary material:**

The online version of this article (doi:10.1007/s00439-016-1640-3) contains supplementary material, which is available to authorized users.

## Introduction

Southeast Asia (SEA) harbours a rich variety of human populations with contrasting patterns of diversity seen in their ethnic cultures, languages, physical appearance and genetic heritage. The population history of this region was traditionally framed in terms of two distinct major prehistoric population movements. The first settlers, described as “Australo-Melanesian” people, arrived around 50–60 ka (thousand years ago) (Barker et al. 2007; Soares et al. 2009), and were the ancestors of several “Australoid” populations found in SEA, New Guinea and Australia (Bellwood 1995; Barker et al. 2007). The second migration occurred during the mid-Holocene (5–4 ka) and involved a large-scale demic expansion of rice agriculturalists starting in South China ~6 ka, which spread in two directions, one towards Mainland Southeast Asia (MSEA), and the other, via Taiwan, to Island Southeast Asia (ISEA), Near and Remote Oceania, and Madagascar (Bellwood 1995, 2005; Gray et al. 2009). Proponents of this “two-layer” model (Bellwood and Dizon 2008), drawn essentially from historical linguistics and some archaeological data, argue that the South Chinese rice agriculturalists partly or largely replaced the previous inhabitants of the region, whilst spreading Austronesian languages in ISEA and Austroasiatic languages in MSEA (Benedict 1976; Bellwood 1995; Bellwood et al. 2006). It is, however, possible that ISEA received direct influence from both of these hypothetical Neolithic migrations, as suggested by Anderson (2005), taking into consideration both archaeological and linguistic evidence. Anderson (2005) offered a more comprehensive view of the Neolithic spread in the region, suggesting that it most likely followed a reticulate pattern, and not a linear expansion model. He proposed the existence of two Neolithic movements from different sources: an earlier minor one ~4.5 ka from MSEA (“Neolithic I”), related to the spread of Austroasiatic languages and basket or cord-marked ceramics, into the Malay Peninsula and Borneo; and a second, major wave (“Neolithic II”), encompassing the hypothetical “out-of-Taiwan” migration (Bellwood and Dizon 2005, 2008). Our recent genetic work supports this view (Soares et al. 2016) but emphasizes that both mid-Holocene expansions were due to small-scale migrations.

Our genetic evidence suggests that other demographic events also contributed to current population structure in SEA, especially as a consequence of the massive climatic changes that occurred at the end of the Last Glacial Maximum (LGM). In the Late Pleistocene, ~20 ka, global sea levels were ~130 m below present-day levels, MSEA and Western ISEA were interconnected by a vast continental landmass, called Sundaland (Barker and Richards 2013), that facilitated early human dispersals through the region (Bird et al. 2005). After the LGM, rapid episodes of sea-level rises at ~14.5, 11.5 and 7.5 ka flooded about half of the land area of Sundaland, with a concomitant doubling of the length of the coastline (Oppenheimer 1998; Bird et al. 2005; Soares et al. 2008). Taking into consideration the past climatic changes in SEA, and the pressure suffered from the flooding of large areas of the landscape, some authors have suggested that these episodes triggered massive migratory events in the region (Oppenheimer 1998; Solheim 2006; Soares et al. 2008). Thus the dispersals across SEA could have resulted from movement and expansion of indigenous Southeast Asian people, possibly reflected in the increase in sites across ISEA at the end of the Pleistocene (O’Connor and Bulbeck 2014). Following this premise, Solheim’s Nusantao Maritime Trading and Communication Network hypothesis (NMTCN) (Solheim 2006) argues that Southeast Asian natives, regardless of language, developed a highly maritime-oriented culture as a result of the changes in the climate and landscape in the region which promoted successful exchange systems between populations in the region for the past 10 ka. The cultural and linguistic similarities could then have been promoted through this wide-ranging trade and communication network.

Recent technological advances have led to the generation of huge amounts of new genetic data. Maternal, paternal and autosomal genetic markers have all been used to shed light on population migration history but genetic studies on SEA and the Pacific are often still framed within the two-layer model. For example, Friedlaender et al. (2008) suggested that the autosomal variation of Remote Pacific Islanders resulted almost solely from the mid-Holocene expansion of Austronesian-speaking Taiwanese, although their analysis did not include SEA populations. From a slightly different premise, Kayser et al. (2008) also argued that the Polynesian populations have clear maternal Asian ancestry, while Y chromosomes are mostly from New Guinean populations. On this view, Polynesian genetic make-up becomes the result of the intermarriage between Austronesian-speaking females carrying Asian mtDNA lineages (e.g. the mtDNA “Polynesian motif”) with male Melanesians en route to the Pacific.

However, although the Polynesian motif (defining mtDNA haplogroup B4a1a1) is extremely frequent in the Remote Pacific, with ancestral lineages present equally in ISEA and Taiwanese aboriginals, this need not imply an “Austronesian dispersal”. In fact, the Polynesian motif itself is absent in most of ISEA and not found further west of Wallace’s line, except for southeast Borneo, and it has a coalescence time much greater than expected if it had emerged en route between Taiwan and the Pacific in the mid-Holocene (Soares et al. 2011). The molecular-clock evidence (strongly corroborated by archaeologically consistent estimates for the entry into Remote Oceania itself) rather suggests the ancestral lineage reached the Pacific in the Early Holocene (Soares et al. 2011), where it evolved into the Polynesian motif ~6–7 ka, probably in the Bismarck Archipelago, before expanding both east into the Remote Pacific and west back into ISEA.

In fact, an increasing number of studies in recent years have indicated that the simple two-layer expansion model does not capture the complexity of the demographic history in ISEA (Bulbeck 2008; Donohue and Denham 2010). Karafet et al. (2010), analysing patterns of Y chromosome variation (Y-SNPs), argued for a discontinuous four-phase colonization process with several population incursions in SEA, starting with the introduction of basal haplogroups with the first settlers, followed by Late Pleistocene/Early Holocene postglacial migrations from the mainland, the mid-Holocene “out-of-Taiwan”, and a more recent migration in the historical era. Significantly, they suggest that only few paternal lineages are associated with the Austronesian dispersal, and that the other major lineages date to earlier population movements.

These results have been corroborated in other recent studies (Trejaut et al. 2014; Soares et al. 2016). In terms of mtDNA, although studies showed the existence of mtDNA lineages shared between Austronesian speakers of Formosan, Filipino and other ISEA populations (Trejaut et al. 2005; Tabbada et al. 2010), many have contradicted a demic “out-of-Taiwan” expansion due to the time frame (Trejaut et al. 2005; Hill et al. 2007; Soares et al. 2008, 2016). Moreover, some ISEA maternal mtDNA lineages did not trace back their origin to Taiwan, but instead arose within the ISEA region and spread toward Taiwan, probably because of climatic changes (Soares et al. 2008, 2016). For example, mtDNA haplogroup E underwent major expansions and dispersals in the Early to mid-Holocene, extending west into Malaysia, east into New Guinea and north into Taiwan, somewhere between 8 and 4 ka (Soares et al. 2008) (using the recalibrated mtDNA clock: Soares et al. 2009). Thus Taiwan appears to have been a recipient of haplogroup E lineages from the south, before the Austronesian dispersal, rather than being the major source of Holocene population migrations southwards across ISEA (as in the “out-of-Taiwan” model). Genome-wide analyses have independently supported the notion that Taiwan was, at least in part, the recipient of genetic input from ISEA, rather than the other way around (Abdulla et al. 2009).

Nevertheless, the genetic picture of SEA remains far from being fully understood. Recently, Soares et al. (2016) performed a founder analysis for ISEA that highlighted three major haplogroups representing the main signals in the analysis; two were postglacial or Early Holocene (haplogroups E and B4a1) and one was a mid-Holocene “out-of-Taiwan” marker (haplogroup M7c3c). Overall, the data, representing 30–40 % of all present-day mtDNA lineages, matched the Early Holocene period, implying that although migrations from Taiwan did occur in the mid- to late Holocene, the so-called Austronesian expansion was mainly a process of cultural diffusion and assimilation. The remaining mtDNA lineages, many displaying low frequencies, cannot be so clearly partitioned using a founder analysis based on HVS-I sequences (first hypervariable segment of the control region). Here, therefore, we analyse in detail the sequence variation of whole-mtDNA genomes (“mitogenomes”) of these low frequency mtDNA lineages. These lineages have already been tentatively associated with various demographic events in SEA, including the first settlement (haplogroup F3, R9b), Early Holocene postglacial expansions (haplogroup R9c, N9a) and mid-Holocene dispersals from Taiwan (haplogroups B4c1, F1a4, B5b, Y2, B4b1 and D5) (Hill et al. 2006, 2007; Soares et al. 2016), potentially identifying the spread of Neolithic material culture. We previously analysed R9b with whole mtDNAs (Hill et al. 2006), but the subsequent increase in sampling, as well as a revision of the molecular clock (Soares et al. 2009) demand a reassessment of the phylogeography of the clade. A comprehensive study of these low-frequency haplogroups in Southeast Asia can complete the picture of both the main dispersal routes and the impact of dispersals on the population history in the region. Our study ranges across the vast geographic region of Taiwan, MSEA, ISEA and Near Oceania, in contrast to other recent studies (Tabbada et al. 2010; Loo et al. 2011; Ko et al. 2014), in which more limited geographic regions were targeted.

## Methods

### Population samples and whole-mtDNA sequence analysis

We selected the population samples used in this study on the basis of the information from mtDNA hypervariable segment I (HVS-I), which allowed the broad classification of the samples into haplogroups. We selected 114 samples belonging to 10 haplogroups in the region of Southeast Asia (B4b1; B4c1; B5b, D5; F1a4; F3, N9a; R9b, R9c and Y2). We chose the samples to constitute a dataset representative of the genetic variability of the general population. We included 2 from China, 23 from Taiwan, 61 from MSEA (22 from Vietnam; one from Thailand; 31 from Peninsular Malaysia; 4 from Laos and three from Myanmar (Burma)); 26 from ISEA (12 from island of Borneo—three from Kota Kinabalu (Sabah, Malaysian state), five from Brunei, four from Palangkaraya (Indonesian province Central Kalimantan); 11 from other parts of Indonesia and three from the Philippines), and two from Micronesia (details in Table S1). The work was approved by the University of Huddersfield, SAS Ethics Committee.

For the whole-mtDNA genome sequencing, we followed the methodology and checking procedures reported in Pereira et al. (2010) and we analysed the sequences with BioEdit 7.0.4.1 (Hall 1999) and Sequencher 5.2.3 sequences analysis software (Gene Codes Corporation, Ann Arbour, MI, USA). We deposited 114 new whole-mtDNA sequences in GenBank (Accession Numbers KU521394-KU521507).

To obtain detailed phylogenetic reconstruction and precise age estimates of clades and times of expansion, we took comparative data from the literature. More specifically, we used 829 published whole-mtDNA genomes, which included 52 from MSEA, 197 from Taiwan, 173 from ISEA, and 407 from neighbouring regions (for more detailed information see Table S2).

### Statistical analyses

To avoid any nomenclature conflicts, we followed the criterion of PhyloTree [mtDNA tree Build 16 (19 Feb 2014)] (van Oven and Kayser 2009). We disregarded the transition at 16,519 and the C-length polymorphisms in regions 16,180–16,193 and 309–315 in the analyses (Soares et al. 2009). We performed the classification of the variants with mtDNA GeneSyn (Pereira et al. 2009), and scored mutations in relation to the revised Cambridge reference sequence (rCRS) (Andrews et al. 1999).

We performed preliminary reduced-median network analyses (Bandelt et al. 1995), providing a suggested branching order for the trees, and used these to construct a putative most-parsimonious tree based on the relative mutation rates of the different positions (Soares et al. 2009).

For estimation of the coalescence times for specific clades in the phylogeny, we used the ρ statistic and maximum likelihood (ML). We used the ρ statistic with a mutation-rate estimate for the whole-mtDNA sequence of one transition in every 3624 years, corrected for purifying selection using the calculator developed by Soares et al. (2009), and a synonymous mutation rate of one substitution every 7884 years. We estimated standard errors as in Saillard et al. (2000). We also obtained ML estimates of branch lengths using PAML 4.7a (Yang 1997), assuming the HKY85 mutation model with gamma-distributed rates (approximated by a discrete distribution with 32 categories). We converted mutational distance in ML to time using the whole-mtDNA genome clock described above (Soares et al. 2009).

To access the demographic changes through time in SEA populations associated with the haplogroups studied, we obtained BSPs (Drummond et al. 2005; Fagundes et al. 2008) using BEAST (version 1.7.5) with a relaxed molecular clock (lognormal in distribution across branches and uncorrelated between them) using a mutation rate of 2.6186 × 10^−8^ mutations per site per year for the whole-mtDNA genome (Soares et al. 2012) and the HKY model of nucleotide substitutions with gamma-distributed rates, assuming a generation time of 25 years. In addition, we forced the larger subclades into monophyly to obtain a tree structure that was directly comparable with the remaining analyses (Fagundes et al. 2008). We visualised the plots with Tracer v1.3, and inferred the increment ratio by calculating the number of times that the effective population size increased during specific periods. For a broader overview of the geographic distribution patterns of the lineages, we constructed interpolation maps of spatial frequencies based on their HVS-I sequences (in the range of 16,051–16,400 bp), using the Kriging algorithm of Surfer 8 (Fig. S1).

We estimated founder ages for the main clades present in Taiwan, based on the whole mitogenomes. To minimize the impact of recurrent mutation and back-migrations, we selected founders on the strength of their source diversity, using an *ƒ1* criterion, which stipulates that a sequence type is only considered a founder if it presents at least one derived branch in the source population (Richards et al. 2000). We calculated founder ages and confidence intervals as described before (Richards et al. 2000; Soares et al. 2012; Rito et al. 2013) and plotted the overall pattern at 200-years intervals. Since the current founder analysis methodology does not incorporate a time-dependent molecular clock, we made an approximation for the time scale under study. The mutation rate varied between 1 mutation per 2599 and 2679 years for the point estimates of the estimated founders, so we used an average value of 2639.

## Results

### General patterns of migration and expansion in Island Southeast Asia

**Fig. 1:**
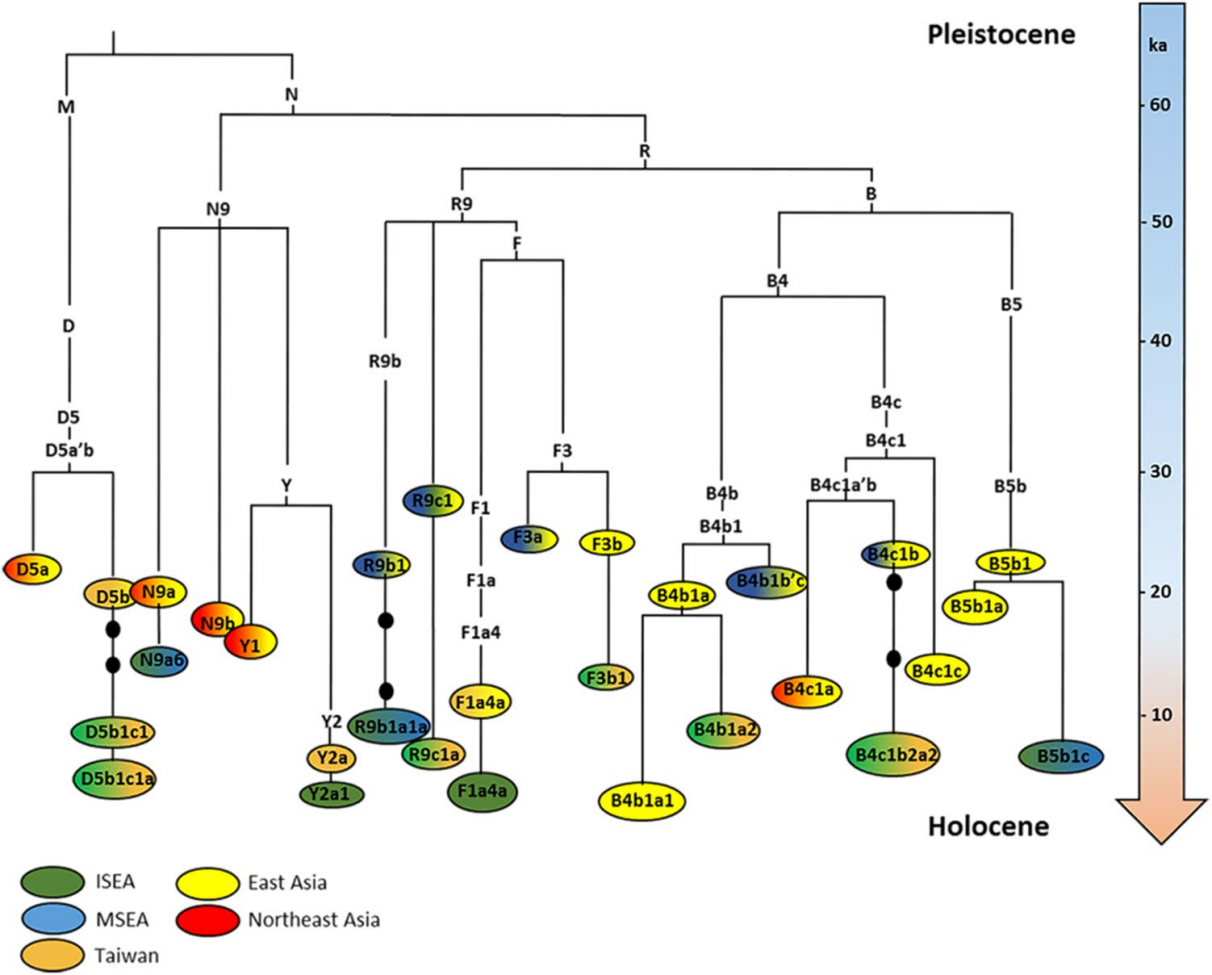
Schematic tree of the subclades most representative in SEA belonging to haplogroups B4b1, B4c1, B5b, D5, F1a4, F3, N9a, R9b, R9c and Y2. The higher-frequency lineages B4a1a, E1, E2 are not shown in the figure, since they were analysed previously by Soares et al. (2016) Tree scaled using maximum likelihood and time-dependent molecular clock for whole-mtDNA genome (in ka). The *shading* represents the geographic distribution of the subclades. Details of age estimates are shown in Table 1

For the phylogeographic analysis, we used 870 previously published and 114 newly sequenced mitogenomes belonging to haplogroups R9b, R9c, F1a4, F3, F4b, B4b1, B4c1, B5b, N9, Y and D5 (Online Resource 1, namely Table S1, Table S2 and Supplementary Note 1, and Online Resource 2). Figure 1 shows an outline topology of the main subclades in East Asia and SEA for these haplogroups, scaled against the ML age estimates (for detail of ρ and ML age estimates, see Table S3). F4b, which entered Taiwan from China at the time of the Neolithic, but does not disperse further into ISEA, is not included. We can group the haplogroups into those with Early Holocene and those with mid-Holocene ancestry in ISEA (Figs. S2 and S3). The clades B4a1, E1, E2 (the higher-frequency lineages analysed previously (Soares et al. 2016), not shown in Fig. 1), F3b1, R9c1a, B5b1c and B4c1b2a2, corresponding to almost 27 % of all present-day mtDNA lineages in ISEA, most probably expanded within ISEA mainly between 10 and 7 ka, many of them reaching Taiwan at some point in the last 8 ka. Haplogroups M7c3c, Y2a1, B4b1a2, F1a4, D5b1c1 and M7b3, amounting to ~20 % overall in ISEA primarily show founder ages that indicate a mid-Holocene, potentially Neolithic entrance into this region, probably from a source in Taiwan (Table 1).

**Table 1:** Age estimates using rho (ρ) and ML for major subclades in ISEA for haplogroups B4b1, B4c1, B5b, D5, F1a4, F3, N9a, R9b, R9c and Y2. Ages and 95 % confidence intervals (CI) in thousands of years

mtDNA lineage	*N*	PAML		Rho			
				Total		Synonymous	
		Age	95 % confidence interval	Age	95 % confidence interval	Age	95 % confidence interval
B4b1	129	25,100	[17,000–33,600]	22,600	[12,600–33,200]	32,600	[9400–55,900]
B4b1a2	89	9300	[6800–11,800]	8700	[6400–11,000]	9100	[5300–12,900]
B4c1b2a	56	14,500	[6400–23,000]	11,200	[3800–19,000]	15,500	[0–31,400]
B4c1b2a2	53	8000	[5600–10,500]	5800	[3500–8100]	7700	[1900–13,600]
B5b	89	29,900	[20,700–39,300]	34,300	[24,300–44,700]	43,500	[24,500–62,500]
B5b1	54	23,900	[13,900–34,300]	27,000	[15,800–38800]	44,700	[19,200–70,300]
B5b1a	15	19,000	[8,800–29,800]	20,500	[9300–32,400]	12,600	[0–25,800]
D5	174	33,300	[24,600–42,200]	34,500	[23,000–46,500]	35,600	[15,500–55,800]
D5b1c1	15	9100	[4000–14,400]	12,300	[4300–20,600]	21,000	[900–41,100]
D5b1c1a	11	6000	[0–13,800]	7400	[1200–13,700]	8600	[0–19,500]
D5b3	40	10,900	[5600–16,400]	9900	[2400–17,700]	17,700	[0–37,100]
F1a4	26	16,300	[7000–25,900]	18,600	[6500–31,400]	11,500	[0–26,100]
F1a4a	25	11,700	[3000–20,900]	10,600	[2600–19,000]	11,700	[0–26,800]
F1a4a1	23	4300	[1800–6800]	5200	[1500–9000]	3900	[800–7100]
F1a4a1a	15	3300	[1300–5300]	3500	[1500–5500]	3700	[1000–6400]
F3	88	31,700	[21,500–42,300]	37,900	[22,900–53,600]	35,300	[12,400–58,400]
F3a	20	26,600	[16,500–37,200]	31,500	[18,700–44,900]	26,400	[6000–46,800]
F3a1	16	16,600	[9000–24,500]	15,600	[9300–22,100]	13,300	[1300–25,300]
F3b	68	25,200	[15,400–35,400]	28,900	[13,600–45,100]	27,800	[3500–52,200]
F3b1	65	12,400	[5200–20,000]	12,000	[4700–19,700]	12,100	[0–25,100]
N9	254	50,600	[37,100–64,600]	38,500	[27,700–49,600]	36,100	[20,700–51,400]
N9a	127	20,000	[14,500–25,500]	17,500	[13,000–22,100]	18,400	[10,900–25,900]
N9a10	18	16,600	[11,000–22,400]	14,600	[8800–20,600]	14,900	[4600–25,200]
N9a10a	9	10,000	[4700–15,400]	9100	[4100–14,200]	14,000	[1600–26,400]
N9a10a1	6	6300	[200–12,500]	5200	[1200–9300]	6600	[0–14,300]
N9a6	45	14,800	[9900–19,800]	12,700	[7100–18,500]	9600	[2900–16,400]
R9b	45	38,700	[23,900–54,300]	32,800	[20,400–45,900]	31,400	[13,600–49,200]
R9b1a	32	18,600	[10,900–26,700]	20,700	[13,200–28,500]	23,900	[9800–38,100]
R9b1a1	18	11,600	[6000–17,300]	13,400	[6600–20,300]	9600	[3800–15,500]
R9b2	4	5700	[1300–10,200]	5200	[1600–8900]	5900	[0–12,600]
R9c1	45	28,500	[17,200–40,300]	25,200	[13,200–37,800]	33,100	[9600–56,600]
R9c1a	33	5900	[3700–8300]	5100	[2600–7700]	6900	[400–13,500]
Y	98	28,000	[16,100–40,500]	24,250	[14,000–34,900]	31,200	[11,600–50,900]
Y2	50	9600	[5000–14,400]	9250	[3100–15,600]	8800	[0–20,100]
Y2a	43	6100	[3200–9100]	6700	[2000–11,500]	8400	[0–21,400]
Y2a1	36	4100	[2300–5900]	4500	[2400–6600]	1500	[400–2700]

The tree topology for each clade reinforces these inferences. The first group (encompassing the higher frequency Early Holocene candidate haplogroups analysed previously by Soares et al. (2016), B4a1a, E1, E2, and the remaining lower-frequency haplogroups analysed in this study, F3b1, R9c1a, B5b1c and B4c1b2a2) displays long branches with a lack of branching nodes, indicating bottleneck/drift between, in general, 20–15 and 10 ka. More specifically, 15.1–9.9 ka from B4a1 to B4a1a (four mutations), 24.0–6.7 ka in B5b1 and B5b1c (seven mutations), 25–6 ka in F3b to F3b1 (six mutations), 14.5–8 ka from B4c1b2a to B4c1b2a2 (two mutations), 28.5–6 ka from R9c1 to R9c1a (eight mutations), and 24–8.3 and 12.7 ka from E to E2 (five mutations) and E1 (four mutations), respectively (Fig. S4). On the other hand, the mid-Holocene “out-of-Taiwan” clades (M7c3c, Y2a1, B4b1a2, F1a4, D5b1c1 and M7b3) show considerably shorter branch lengths separating the clade that expanded within current Austronesian-speaking populations and the ancestral point in continental Asia. The age estimate of the latter is usually within the 12–6 ka range: 11.7–4.3 ka from F1a4a to F1a4a1 (two mutations), 9.6–6.1 from Y2 to Y2a (one mutation), 17.7–9.3 ka in B4b1a + 207 to B4b1a2 (one mutation; but B4b1a2 exists in mainland China and Japan and is probably the founder clade, meaning that no mutation separates the Asian ancestor and the ISEA clade), 11.8–5.3 ka from M7c3 to M7c3c (one mutation) and 7.9–5.7 from M7b3 to M7b3a (one mutation) (Fig. S4).

A plausible explanation for this pattern may lie in the fact that the time interval before 8 ka was very likely a period of low effective population size in ISEA, with expansions largely restricted to the last 10 ka. The haplogroups that probably expanded in the Early Holocene postglacial period could therefore represent clades that were already present in ISEA by the time of the flooding of the Sunda shelf, and were maintained during the low effective population size period, and later expanded ~10–7 ka. On the other hand, the candidate mid-Holocene “out-of-Taiwan” clades were present in mainland China by 10 ka and moved to Taiwan and ISEA in the last 7–4 ka; and they do not display similar long branches but only branches with one or two mutations linking them to the mainland ancestor.

This is also reflected in the BSPs (Bayesian skyline plots, Fig. S5), which display changes in effective population size over time. No increment in population size is observed previous to 10 ka except at ~60 ka, which reflects mainly the basal M, N and R “out-of-Africa” founder nodes and occurred outside ISEA (Macaulay et al. 2005). Most of the clades analysed in the BSP (except maybe for haplogroups E and F3b) were not present in ISEA before 20–15 ka. From about 10.5–8 ka, a 19-fold increment in the population size occurred, followed by a 70-fold increase in the mid-Holocene, starting ~4 ka (Table 2). The BSP containing the clades analysed here (as well as all B4a, M7 and E clades) indicates two time periods of population expansion that perfectly fit the time of expansions inferred from the phylogeographic analysis.

**Table 2:** Peaks of population size through time as obtained from BSPs for all haplogroups examined in this study, and the overall data for ISEA

Data set	Peak (ka)	Range of increment (ka)	Increment ratio
Haplogroup			
N9	11.9	9.8–14.9	0.5
	6.8	3.8–7.7	1.8
B4b1	6.1	4.9–7.0	2
	0.8	0–2.0	6
B4c1	6.1	5.0–7.6	2.5
Y2	3.1	2.2–4.2	1.1
F3	7.4	5.2–10.3	0.1
	2.5	4.1–1.2	1.1
R9b	5.4	0.9–7.1	1.5
B5b	16.8	15.8–18.9	0.7
	3.9	3.3–5.0	7.9
D5	7.7	3.5–13.1	2.1
R9c	3.6	2.1–5.2	1.7
F1a4	2.4	1.7–4.0	0.3
Region			
ISEA	9.0	8.2–10.6	10.1
	2.6	2.3–4.1	76.9

It is worth emphasizing that the above mentioned increments correspond to clade expansions, which do not represent their relative frequencies in the ISEA population—for example, the low frequency clades analysed here are actually oversampled in relation to the more common clades. Furthermore, more ancient basal clades from SEA, namely basal M clades (Hill et al. 2007), were not included, and these could well have expanded in both periods.

### Founder mtDNAs in Taiwan

**Fig. 2:**
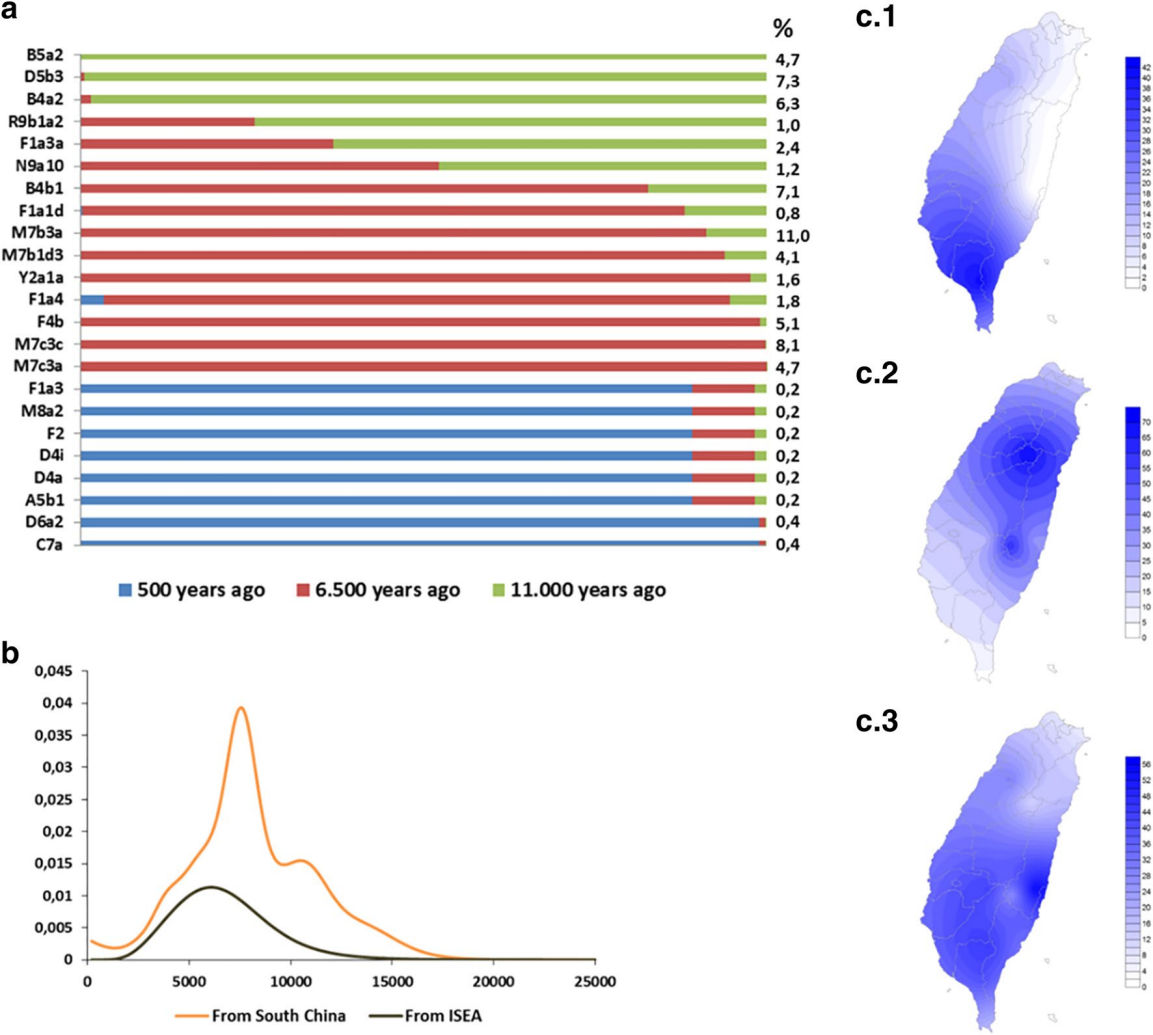
Analysis of maternal genetic flow into Taiwan. **a** Probabilistic distribution of founders from mainland Asia, assuming three migrations, using *ƒ1* criterion; **b** scan of migration time into Taiwan from South China (*orange line*) and ISEA (*black line*). **c** Frequency distribution maps of Taiwan based on HVS-I data: *c1* Pooled frequency of candidate postglacial mainland South China haplogroups (B5a2, B4a2, D5b3 and R9b1a2); *c2* Pooled frequency of candidate Neolithic South China haplogroups (B4b1a2, F1a4, M7c3, Y2a, F4b, N9a10, M7b3a and M7b1d3); *c3* Pooled frequency of candidate ISEA influx haplogroups (B4a1a, B5b1c, F3b1a, B4c1b2a2, E1 and E2). The map of Taiwan was adapted under the terms of the GNU Free Documentation License

We separated founders entering Taiwan into lineages that had a clear mainland origin in the last 15 ka and those that probably expanded in ISEA and Taiwan in the Early Holocene period from a source in ISEA (Table S5 and Fig. 2a). The latter generated a single peak at just above 6 ka (line in black in Fig. 2b). The clades for which we postulated a mainland origin led to a pattern that showed two peaks, one at 7.6 ka and another at 11 ka. These could be, respectively, correlated with the entry of rice agriculturalists, in accordance with the “out-of-Taiwan” model, and to arrivals in the postglacial Early Holocene period (or simply marking the separation of continental China and Taiwan due to sea-level rises). We further performed the analysis by stipulating two points of migration, one at 6.5 ka (following the “out-of-Taiwan” model) and one at 11 ka, to allocate each clade probabilistically to a migration event.

Four of the clades, B5a2, B4a2, D5b3 and R9b1a2, were probably present in Taiwan for more than 10 ka. All the other clades analysed (B4b1a2, F1a4, M7c3, Y2a, N9a10, M7b3a and M7b1d3) showed a higher probability of an entry with the Neolithic material culture (Fig. 2a). An important feature is that all the clades that we detected as “out-of-Taiwan” in the phylogeographic analysis were present here as input from the mainland dating to the spread of the rice Neolithic. This indicates two things: firstly, it provides further support to identify these clades as “out-of-Taiwan” candidates; but it also suggests that (at least until the “out-of-Taiwan” migration into ISEA) there was some level of separation of the expanding population from the autochthonous population of Taiwan, since the more ancient clades in Taiwan, as well as the ones entering before this time from ISEA, do not show any evidence of playing a role in the “out-of-Taiwan” migration.

We have further studied the distribution of the clades in the Taiwanese aboriginal populations according to their ancestry (Fig. 2c). Clades from China that show an Early Holocene ancestry in Taiwan are much more frequent in the south and almost absent on the east coast of Taiwan (Fig. 2c.1). The clades that we infer to have entered Taiwan carried by rice-agriculturalists from South China are much more frequent in the northern tribes and east coast of Taiwan (Fig. 2c.2). The influx clades that are of Early Holocene age in ISEA are much more evenly distributed across the extant aboriginal groups of Taiwan, with a peak on the east coast and a lower frequency in the northern tribes (Fig. 2c.3).

### Genetic ancestry of the “out-of-Taiwan” clades

**Fig. 3:**
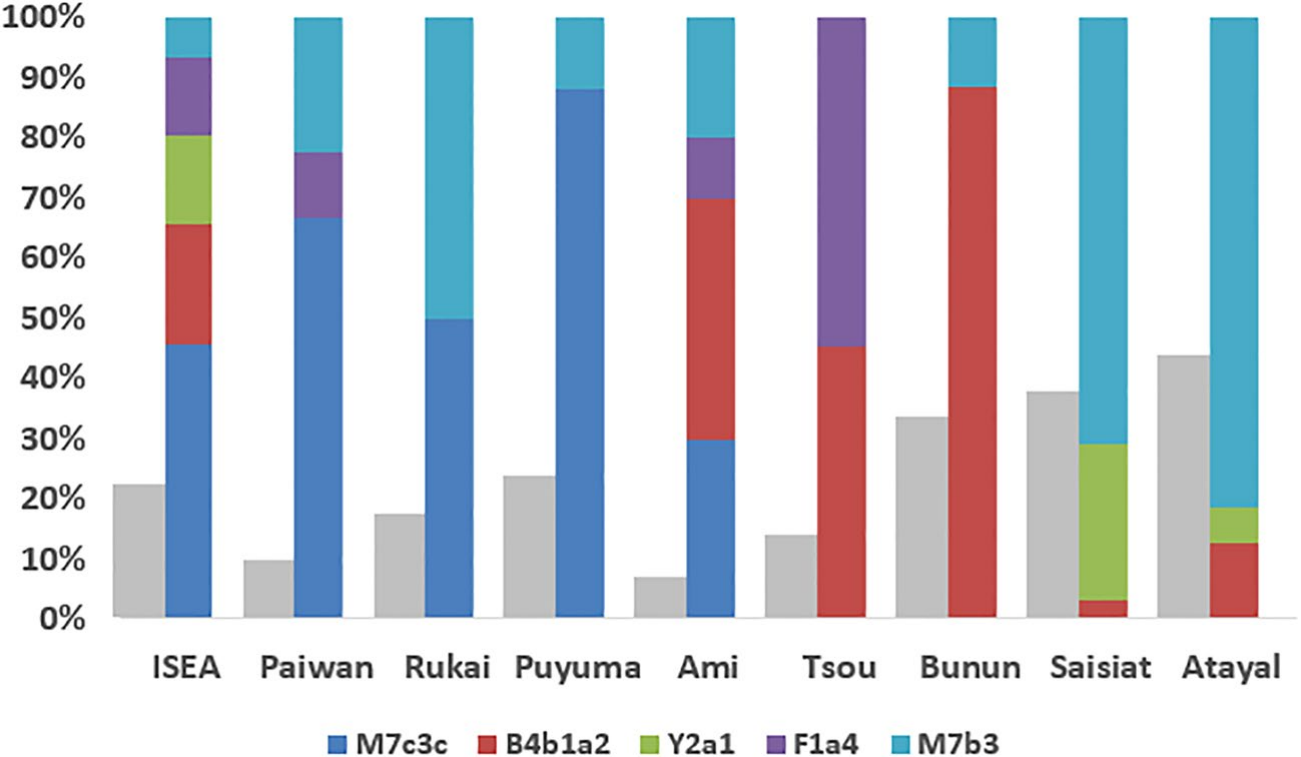
Estimated contributions of Taiwanese “out-of-Taiwan” mtDNA lineages in the ISEA and Taiwanese aboriginals gene pool. The *grey bar* represents the overall frequency of those lineages in each population and the *second bar* represents the relative frequency of those haplogroups within each population

Since all the aboriginal groups now carry some clades that were involved in the “out-of-Taiwan” event (Fig. 3), we checked the best matches between this “out-of-Taiwan” composition and relative frequencies of those clades in the Taiwanese aboriginal groups, in order to provide some insights into the origin of the expanding population from Taiwan. Although northern tribes are the only ones that contain haplogroup Y2a, they lack the major “out-of-Taiwan” clade, M7c3c but carry a very high frequency of M7b3, which is a minor clade in ISEA. Most probably, considering the drift that the Taiwanese aboriginal groups have undergone (due to their small size and isolation) in the last few thousand years, Y2a was probably lost due to drift at other locations in Taiwan. The southern groups show the highest frequency of M7c3c, the major mid-Holocene “out-of-Taiwan” marker, but as well as Y2a they also lack B4b1, the second most common “out-of-Taiwan” clade in ISEA. B4b1 and F1a4 are more common in the central Taiwan tribes, but the overall composition that more closely matches the inferred group that dispersed into ISEA is that of the Ami of the east coast. They show a relatively high frequency of M7c3c and B4b1, the two major “out-of-Taiwan” clades in ISEA, and also carry M7b3 and F1a4. Curiously, the overall frequency of clades entering Taiwan at 6.5 ka in the founder analysis was higher for the northern tribes (such as Saisiat), as well as the Ami. Therefore the genetic evidence is concordant with the linguistic evidence suggesting that the Ami language is the closest Formosan candidate to the Malayo-Polynesian branch of the Austronesian family (Ross 2005).

## Discussion

Vigorous debate continues among archaeologists, linguists and geneticists as to which major population movements shaped the demographic history of Southeast Asia. Here we tested a number of human mtDNA clades, individually present at low frequency, but together amounting to a quarter of the ISEA maternal component, and which had previously been suggested as representing various different stages of the genetic history of the region. We have shown that the phylogeography of these low-frequency clades closely matches two major hypothetical events, each one already recognised from the more frequent clades: namely, postglacial expansions in the Early Holocene and an “out-of-Taiwan” dispersal in the mid-Holocene. This study therefore complements our earlier work on the more frequent B4a1a, E and M7 clades, which represent about a third of the mtDNA lineages in ISEA.

At the end of the LGM, SEA underwent a major environmental change, with rising sea levels flooding many low-lying areas of the Sunda shelf, eventually creating the modern coastline (Bird et al. 2005). The loss of almost half of the land area and the environmental transformations, with the replacement by rainforest of the savannah and monsoon forest to which the Sunda populations had adapted, is thought to have caused enormous population displacements and cultural changes (Bird et al. 2005; Solheim 2006; Soares et al. 2008; Tumonggor et al. 2013). The effective population size is likely to have been low during this cataclysmic period. We would also expect that the later expanding populations would be composed of an unrepresentative sample of the maternal pool of the Sunda populations that existed from 20 to 10 ka. From our analysis, the clades that likely expanded in the Early Holocene postglacial have three basic characteristics: a split time with continental Asia of at least 15 ka; a lack of ancestral branching nodes between 15 and 8 ka; and, of course, an estimate of the age of expansion of the clade mostly between 10 and 7 ka. Haplogroups B4a1a, B5b1c, F3b1, B4c1b2a2, R9c1a and E fit this pattern well; and their BSPs also show lower effective population sizes until about 10 ka ago. Undoubtedly, many lineages from ISEA must have been lost during that period, potentially explaining the lack of ancestral lineages, such as the ancient DNA E1 lineage detected further north (Ko et al. 2014) when the overall pattern of haplogroup E strongly suggests ISEA as the point of origin and evolution for the clade (Soares et al. 2008) and the age estimate based on the general mtDNA clock and on ancient DNA calibration places the clade within ISEA much before the putative migration of rice agriculturists into Taiwan (Soares et al. 2016).

**Fig. 4:**
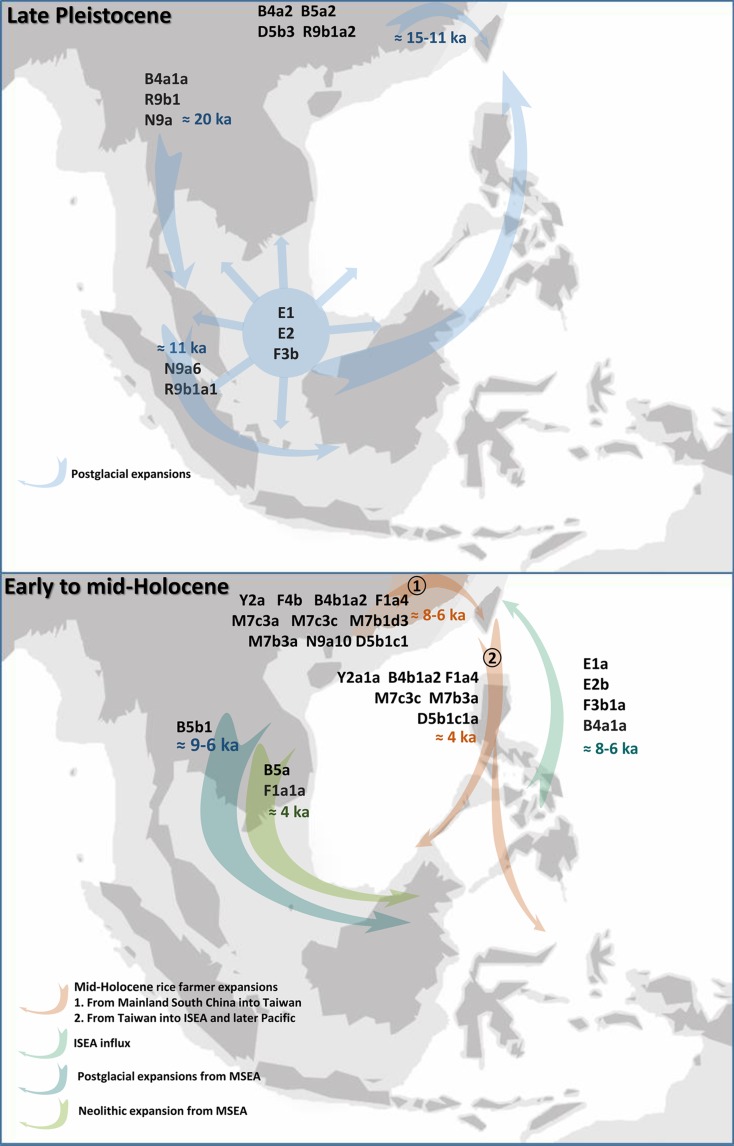
Outline of maternal lineages involved in the main human migrations in the region of Southeast Asia and Taiwan. Includes those discussed here and also those described previously in Soares et al. (2016), including B5a and F1a1a, which were inferred to have dispersed from MSEA with the Neolithic. *Dark shading* represents the modern coastlines and the extent of Sundaland at the LGM is represented by the *light shading*. The map was obtained from the website http://www.outline-world-map.com

The observed two-way Early Holocene population expansions between East and Southeast Asia probably did not take place as a single monolithic event, but in multiple radiations from 10 ka onwards [cf. the “early train” hypothesis (Jinam et al. 2012)]. Eventually, some of the lineages reached Taiwan. The founder peak indicated a period ~ 6 ka, but ISEA lineages might have reached the island at several times in the postglacial period. The frequency patterns of these lineages in Taiwan clearly display a south-to-north cline as expected from lineages arriving from ISEA (Fig. 4).

A founder analysis from China into Taiwan showed that part of Taiwan’s aboriginal maternal gene pool also included lineages that were present there for more than 10 ka (Chang 1989; Olsen and Miller-Antonio 1992). Some of them could be lineages that were present in the population of that part of the Asian continent before sea-level rise separated Taiwan from the mainland. Consistent with this, these ancient lineages are present throughout all the Taiwanese tribes.

In the last 7 ka, ISEA was not the only source of gene flow into Taiwan. Dispersals from South China also occurred in this time frame, and according to the prevailing “out-of-Taiwan” model these individuals were rice agriculturalists. The “out-of-Taiwan” model states that Neolithic farmers who had settled in Taiwan from South China around 7–6 ka, amongst whom the Austronesian languages arose, spread south after ~4.5 ka into the Philippines, Indo-Malaysia and into the Pacific, carrying with them the Neolithic package and the Proto-Malayo-Polynesian language (Ko et al. 2014). Here we see that B4b1a2, F1a4a, Y2a1 and the very minor clade D5b1c1 each show a strong signal of Taiwanese Neolithic ancestry in ISEA, forming—together with M7c3c and M7b3—the bulk of the “out-of-Taiwan” ancestry in ISEA (Fig. 4).

We can therefore refine our estimate of the fraction of Taiwanese Neolithic lineages present in ISEA today. It is theoretically possible that the frequency of mid-Holocene “out-of-Taiwan” founders into ISEA might be slightly underestimated as some of the founders that entered Taiwan from ISEA might have back-migrated to ISEA in the mid-Holocene. However, our results showed no evidence that pre-existing autochthonous lineages in Taiwan were involved in the mid-Holocene “out-of-Taiwan” migration, suggesting that there was a degree of isolation between those ancient lineages and the ones expanding from South China. Given this, and considering that we did not detect any putative mid-Holocene founders within the mitogenome tree of B4a1a, E1, E2 and F3b, it seems likely that these lineages, as the autochthonous lineages in Taiwan, were isolated from the putative “out-of-Taiwan” migrating population. Overall, our ISEA database (2117 samples from the Philippines and Indonesia) includes 19.5 % Neolithic lineages. The overall value is not very meaningful, however, because the estimate varies considerably from region to region. As one would expect from the inferred pattern of spread of the Malayo-Polynesian languages, the value is highest in the Philippines, where it amounts to about 28 % of extant lineages, and next in Eastern Indonesia (Sulawesi and the Lesser Sundas), at 19.7 %, falling to 13.6 % in Western Indonesia (Java, Sumatra and Bali) and only 10.3 % in Kalimantan. It is possible that the small groups of dispersing Malayo-Polynesian speakers assimilated indigenous populations in the Philippines before spreading further south and east, although this “demic diffusion” model seems not to apply (despite a longer time frame) within Taiwan.

There are several hints of an increase in population size in the archaeological record after 4 ka that might reflect the expansion of both autochthonous and immigrant groups. First, cave sites with evidence of occupation up to 4 ka, such as Niah Cave in Sarawak and Ulu Leang 1, Leang Burung 1 and Leang Tuwo Mane’e in Sulawesi, also generally show evidence of continued occupation after that date. Second, many cave sites register their initial evidence for occupation between 4 and 3 ka; these include Torongan Cave and Reranum Cave in the Batanes Islands in the Philippines, Arku Cave in Luzon, Bukit Tengkorak in Sabah, Leang Karassak in Sulawesi and Uattamdi in the Moluccas. Third, a new class of sites comprising dated open sites is evident after 4 ka, represented for instance by Dimolit in Luzon and the Kalumpang sites in Sulawesi. There is an increasing number of these sites after 2 ka, for instance the Buni sites in northwest Java, Gilimanuk and Pacung/Sembiran in Bali, and Melolo in Sumba (Bellwood 1997; Bulbeck et al. 2000; Bellwood and Dizon 2014).

Mid-Holocene input into Taiwan from the mainland is also very clear in the founder analysis, and it is especially notable that all of the lineages that showed an “out-of-Taiwan” ancestry in ISEA are also Neolithic markers for the settlement of Taiwan from South China. Although the frequency of these lineages throughout the various aboriginal groups was never greater than 50 % (except in the Ami, with 56 %), it appears therefore that the admixture that generated the current maternal gene structure of Taiwanese tribes took place progressively after the “out-of-Taiwan” expansion. At the time the “out-of-Taiwan” dispersal occurred, the expanding population seems to have remained distinct, with a full South Chinese ancestry (similar to what also seems to have happened to a considerable degree in Central Europe (Haak et al. 2010) and in the Great Lakes region during the Bantu expansion (Gomes et al. 2015; Silva et al. 2015). Whether the subsequent admixture with autochthonous groups (and, presumably, the assimilation of the language) was rapid or protracted we cannot assess from contemporary data alone, in the absence of ancient DNA evidence. Although some hierarchical ancestry for Austronesian languages have been proposed, the most widely accepted Austronesian tree indicates ten basal branches, including Malayo-Polynesian, which on the face of it might support a simultaneous expansion of this hypothetical Austronesian-speaking agriculturalist population across Taiwan and ISEA. However, since the Ami showed the closest similarity to the ancestral Austronesian-speaking population, an expansion across the island from the east coast from the group of Neolithic pioneers who remained on Taiwan might be suggested.

In conclusion, despite the extensive genetic drift, we have shown that a remarkably consistent picture of prehistoric dispersals can be reconstructed from modern mitogenome patterns. By accounting for all of the rarer lineages in ISEA that have a Holocene Taiwanese ancestry, we are now able to provide a definitive, precise description of Holocene maternal ancestry across Taiwan and Southeast Asia. More generally, the success of this procedure illustrates what we believe to be a valuable approach to the study of human dispersals and settlement using mtDNA data: firstly, a founder analysis using large numbers of control-region sequences; followed by the testing of the time depth of individual candidate founder clusters by the more precise means of whole mitogenomes. This procedure is also amenable to use with other non-recombining marker systems, and ultimately, where feasible, to further testing against ancient DNA.

## Electronic supplementary material

Below is the link to the electronic supplementary material.

Supplementary material 1 (PDF 1617 kb)

Supplementary material 2 (XLSX 542 kb)

## References

[CR1] Abdulla MA, Ahmed I, Assawamakin A, Bhak J, Brahmachari SK, Calacal GC, Chaurasia A, Chen CH, Chen J, Chen YT, Chu J, Cutiongco-de la Paz EMC, De Ungria MCA, Delfin FC, Edo J, Fuchareon S, Ghang H, Gojobori T, Han J, Ho SF, Hoh BP, Huang W, Inoko H, Jha P, Jinam TA, Jin L, Jung J, Kangwanpong D, Kampuansai J, Kennedy GC, Khurana P, Kim HL, Kim K, Kim S, Kim WY, Kimm K, Kimura R, Koike T, Kulawonganunchai S, Kumar V, Lai PS, Lee JY, Lee S, Liu ET, Majumder PP, Mandapati KK, Marzuki S, Mitchell W, Mukerji M, Naritomi K, Ngamphiw C, Niikawa N, Nishida N, Oh B, Oh S, Ohashi J, Oka A, Ong R, Padilla CD, Palittapongarnpim P, Perdigon HB, Phipps ME, Png E, Sakaki Y, Salvador JM, Sandraling Y, Scaria V, Seielstad M, Sidek MR, Sinha A, Srikummool M, Sudoyo H, Sugano S, Suryadi H, Suzuki Y, Tabbada KA, Tan A, Tokunaga K, Tongsima S, Villamor LP, Wang E, Wang Y, Wang H, Wu JY, Xiao H, Xu S, Yang JO, Shugart YY, Yoo HS, Yuan W, Zhao G, Zilfalil BA (2009). Mapping human genetic diversity in Asia. Science.

[CR2] Anderson A (2005). Crossing the Luzon Strait: archaeological chronology in the Batanes Islands, Philippines and the regional sequence of Neolithic dispersal. J Austrone Stud.

[CR3] Andrews RM, Kubacka I, Chinnery PF, Lightowlers RN, Turnbull DM, Howell N (1999). Reanalysis and revision of the Cambridge reference sequence for human mitochondrial DNA. Nat Genet.

[CR4] Bandelt H-J, Forster P, Sykes BC, Richards MB (1995). Mitochondrial portraits of human populations using median networks. Genetics.

[CR5] Barker G, Richards MB (2013). Foraging–farming transitions in Island Southeast Asia. J Archaeol Method Th.

[CR6] Barker G, Barton H, Bird M, Daly P, Datan I, Dykes A, Farr L, Gilbertson D, Harrisson B, Hunt C, Higham T, Kealhofer L, Krigbaum J, Lewis H, McLaren S, Paz V, Pike A, Piper P, Pyatt B, Rabett R, Reynolds T, Rose J, Rushworth G, Stephens M, Stringer C, Thompson J, Turney C (2007). The ‘human revolution’ in lowland tropical Southeast Asia: the antiquity and behavior of anatomically modern humans at Niah Cave (Sarawak, Borneo). J Hum Evol.

[CR7] Bellwood P (1995) Austronesian prehistory in Southeast Asia: homeland, expansion and transformation. In: Bellwood P, Fox JJ, Tryon D (eds) The Austronesians. ANU, Canberra, pp 96–111

[CR8] Bellwood P (1997). The prehistory of the Indo-Pacific archipelago.

[CR9] Bellwood P (2005). First farmers.

[CR10] Bellwood P, Dizon E (2005). The Batanes Archaeological Project and the “Out of Taiwan” hypothesis for Austronesian dispersal. J Austron Stud.

[CR11] Bellwood P, Dizon E, Sanchez-Mazas A, Blench R, Ross M, Peiros P, Lin M (2008). Austronesian cultural origins. Out of Taiwan, via the Batanes Islands, and onwards to western Polynesia. Past human migrations in East Asia matching archeology, linguistics and genetics.

[CR12] Bellwood P, Dizon E (2014). 4000 years of migration and cultural exchange (Terra Australis 40): the archaeology of the Batanes Islands, Northern Philippines.

[CR13] Bellwood P, Fox JJ, Tryon D (2006). The Austronesians: historical and comparative perspectives.

[CR14] Benedict PK (1976). Austro-Thai and Austroasiatic. Ocean Linguist.

[CR15] Bird MI, Taylor D, Hunt C (2005). Palaeoenvironments of insular Southeast Asia during the Last Glacial Period: a savanna corridor in Sundaland?. Quat Sci Rev.

[CR16] Bulbeck D (2008). An integrated perspective on the Austronesian diaspora: The switch from cereal agriculture to maritime foraging in the colonisation of Island Southeast Asia. Aust Archaeol.

[CR17] Bulbeck D, Pasqua M, Di Lello A (2000). Culture history of the Toalean of south Sulawesi, Indonesia. Asian Perspect.

[CR18] Chang K-C (1989). The Neolithic Taiwan Strait. Kaogu.

[CR19] Donohue M, Denham T (2010). Farming and language in Island Southeast Asia. Curr Anthropol.

[CR20] Drummond AJ, Rambaut A, Shapiro B, Pybus OG (2005). Bayesian coalescent inference of past population dynamics from molecular sequences. Mol Biol Evol.

[CR21] Fagundes NJ, Kanitz R, Eckert R, Valls A, Bogo MR, Salzano FM, Smith DG, Silva WA, Zago MA, Ribeiro-dos-Santos AK (2008). Mitochondrial population genomics supports a single pre-Clovis origin with a coastal route for the peopling of the Americas. Am J Hum Genet.

[CR22] Friedlaender JS, Friedlaender FR, Reed FA, Kidd KK, Kidd JR, Chambers GK, Lea RA, Loo JH, Koki G, Hodgson JA, Merriwether DA, Weber JL (2008). The genetic structure of Pacific Islanders. PLoS Genet.

[CR23] Gomes V, Pala M, Salas A, Alvarez-Iglesias V, Amorim A, Gomez-Carballa A, Carracedo A, Clarke DJ, Hill C, Mormina M, Shaw MA, Dunne DW, Pereira R, Pereira V, Prata MJ, Sanchez-Diz P, Rito T, Soares P, Gusmao L, Richards MB (2015). Mosaic maternal ancestry in the Great Lakes region of East Africa. Hum Genet.

[CR24] Gray RD, Drummond AJ, Greenhill SJ (2009). Language phylogenies reveal expansion pulses and pauses in Pacific settlement. Science.

[CR25] Haak W, Balanovsky O, Sanchez JJ, Koshel S, Zaporozhchenko V, Adler CJ, Der Sarkissian CS, Brandt G, Schwarz C, Nicklisch N, Dresely V, Fritsch B, Balanovska E, Villems R, Meller H, Alt KW, Cooper A (2010). Ancient DNA from European early Neolithic farmers reveals their Near Eastern affinities. PLoS Biol.

[CR26] Hall TA (1999) BioEdit: a user-friendly biological sequence alignment editor and analysis program for Windows 95/98/NT. In: Nucleic Acids Symposium Series, vol 41, pp 95–98

[CR27] Hill C, Soares P, Mormina M, Macaulay V, Meehan W, Blackburn J, Clarke D, Raja JM, Ismail P, Bulbeck D, Oppenheimer S, Richards M (2006). Phylogeography and ethnogenesis of aboriginal Southeast Asians. Mol Biol Evol.

[CR28] Hill C, Soares P, Mormina M, Macaulay V, Clarke D, Blumbach PB, Vizuete-Forster M, Forster P, Bulbeck D, Oppenheimer S, Richards M (2007). A mitochondrial stratigraphy for Island Southeast Asia. Am J Hum Genet.

[CR29] Jinam TA, Hong LC, Phipps ME, Stoneking M, Ameen M, Edo J, Saitou N (2012). Evolutionary history of continental Southeast Asians: “Early train” hypothesis based on genetic analysis of mitochondrial and autosomal DNA data. Mol Biol Evol.

[CR30] Karafet TM, Hallmark B, Cox MP, Sudoyo H, Downey S, Lansing JS, Hammer MF (2010). Major east-west division underlies Y chromosome stratification across Indonesia. Mol Biol Evol.

[CR31] Kayser M, Choi Y, van Oven M, Mona S, Brauer S, Trent RJ, Suarkia D, Schiefenhovel W, Stoneking M (2008). The impact of the Austronesian expansion: evidence from mtDNA and Y chromosome diversity in the Admiralty Islands of Melanesia. Mol Biol Evol.

[CR32] Ko AM-S, Chen C-Y, Fu Q, Delfin F, Li M, Chiu H-L, Stoneking M, Ko Y-C (2014). Early Austronesians: into and out of Taiwan. Am J Hum Genet.

[CR33] Loo J-H, Trejaut JA, Yen J-C, Chen Z-S, Lee C-L, Lin M (2011). Genetic affinities between the Yami tribe people of Orchid Island and the Philippine Islanders of the Batanes archipelago. BMC Genet.

[CR34] Macaulay V, Hill C, Achilli A, Rengo C, Clarke D, Meehan W, Blackburn J, Semino O, Scozzari R, Cruciani F, Taha A, Shaari NK, Raja JM, Ismail P, Zainuddin Z, Goodwin W, Bulbeck D, Bandelt HJ, Oppenheimer S, Torroni A, Richards M (2005). Single, rapid coastal settlement of Asia revealed by analysis of complete mitochondrial genomes. Science.

[CR35] O’Connor S, Bulbeck D (2014) Homo sapiens societies in Indonesia and South-Eastern Asia. The Oxford Handbook of the Archaeology and Anthropology of Hunter-Gatherers. Oxford University Press, Oxford, pp 346–367

[CR36] Olsen JW, Miller-Antonio S (1992) The Palaeolithic in southern China. Asian Perspect 31:129–160

[CR37] Oppenheimer S (1998). Eden in the east: the drowned continent of Southeast Asia.

[CR38] Pereira L, Freitas F, Fernandes V, Pereira JB, Costa MD, Costa S, Maximo V, Macaulay V, Rocha R, Samuels DC (2009). The diversity present in 5140 human mitochondrial genomes. Am J Hum Genet.

[CR39] Pereira L, Silva NM, Franco-Duarte R, Fernandes V, Pereira JB, Costa MD, Martins H, Soares P, Behar DM, Richards MB, Macaulay V (2010). Population expansion in the North African late Pleistocene signalled by mitochondrial DNA haplogroup U6. BMC Evol Biol.

[CR40] Richards M, Macaulay V, Hickey E, Vega E, Sykes B, Guida V, Rengo C, Sellitto D, Cruciani F, Kivisild T, Villems R, Thomas M, Rychkov S, Rychkov O, Rychkov Y, Golge M, Dimitrov D, Hill E, Bradley D, Romano V, Cali F, Vona G, Demaine A, Papiha S, Triantaphyllidis C, Stefanescu G, Hatina J, Belledi M, Di Rienzo A, Novelletto A, Oppenheim A, Norby S, Al-Zaheri N, Santachiara-Benerecetti S, Scozari R, Torroni A, Bandelt HJ (2000). Tracing European founder lineages in the Near Eastern mtDNA pool. Am J Hum Genet.

[CR41] Rito T, Richards MB, Fernandes V, Alshamali F, Cerny V, Pereira L, Soares P (2013). The first modern human dispersals across Africa. PLoS One.

[CR42] Ross M (2005). The Batanic languages in relation to the early history of the Malayo-Polynesian subgroup of Austronesian. J Austron Stud.

[CR43] Saillard J, Forster P, Lynnerup N, Bandelt H-J, Nørby S (2000). mtDNA variation among Greenland Eskimos: the edge of the Beringian expansion. Am J Hum Genet.

[CR44] Silva M, Alshamali F, Silva P, Carrilho C, Mandlate F, Jesus Trovoada M, Cerny V, Pereira L, Soares P (2015). 60,000 years of interactions between Central and Eastern Africa documented by major African mitochondrial haplogroup L2. Sci Rep.

[CR45] Soares P, Trejaut JA, Loo JH, Hill C, Mormina M, Lee CL, Chen YM, Hudjashov G, Forster P, Macaulay V, Bulbeck D, Oppenheimer S, Lin M, Richards MB (2008). Climate change and postglacial human dispersals in Southeast Asia. Mol Biol Evol.

[CR46] Soares P, Ermini L, Thomson N, Mormina M, Rito T, Röhl A, Salas A, Oppenheimer S, Macaulay V, Richards MB (2009). Correcting for purifying selection: an improved human mitochondrial molecular clock. Am J Hum Genet.

[CR47] Soares P, Rito T, Trejaut J, Mormina M, Hill C, Tinkler-Hundal E, Braid M, Clarke DJ, Loo J-H, Thomson N, Denham T, Donohue M, Macaulay V, Lin M, Oppenheimer S, Richards MB (2011). Ancient voyaging and Polynesian origins. Am J Hum Genet.

[CR48] Soares P, Alshamali F, Pereira JB, Fernandes V, Silva NM, Afonso C, Costa MD, Musilova E, Macaulay V, Richards MB, Cerny V, Pereira L (2012). The expansion of mtDNA haplogroup L3 within and out of Africa. Mol Biol Evol.

[CR49] Soares P, Trejaut JA, Rito T, Cavadas B, Hill C, Eng KK, Mormina M, Brandão A, Fraser RM, Wang T-Y, Loo J-H, Snell C, Ko T-M, Amorim A, Pala M, Macaulay V, Bulbeck D, Wilson JF, Gusmão L, Pereira L, Oppenheimer S, Lin M, Richards MB (2016) Resolving the ancestry of Austronesian-speaking populations. Hum Genet. doi:10.1007/s00439-015-1620-z 10.1007/s00439-015-1620-z PMC4757630 26781090

[CR50] Solheim WG (2006). Archaeology and culture in Southeast Asia: unraveling the Nusantao.

[CR51] Tabbada KA, Trejaut J, Loo JH, Chen YM, Lin M, Mirazon-Lahr M, Kivisild T, De Ungria MC (2010). Philippine mitochondrial DNA diversity: a populated viaduct between Taiwan and Indonesia?. Mol Biol Evol.

[CR52] Trejaut JA, Kivisild T, Loo JH, Lee CL, He CL, Hsu CJ, Lee ZY, Lin M (2005). Traces of archaic mitochondrial lineages persist in Austronesian-speaking Formosan populations. PLoS Biol.

[CR53] Trejaut JA, Poloni ES, Yen J-C, Lai Y-H, Loo J-H, Lee C-L, He C-L, Lin M (2014). Taiwan Y-chromosomal DNA variation and its relationship with Island Southeast Asia. BMC Genet.

[CR54] Tumonggor MK, Karafet TM, Hallmark B, Lansing JS, Sudoyo H, Hammer MF, Cox MP (2013). The Indonesian archipelago: an ancient genetic highway linking Asia and the Pacific. J Hum Genet.

[CR55] van Oven M, Kayser M (2009). Updated comprehensive phylogenetic tree of global human mitochondrial DNA variation. Hum Mutat.

[CR57] Yang Z (1997). PAML: a program package for phylogenetic analysis by maximum likelihood. CABIOS.

