# Supplementary material for: Quantifying the legacy of the Chinese Neolithic on the maternal genetic heritage of Taiwan and Island Southeast Asia
Source: Hum Genet. 2016 Feb 13;135(4):363–76. doi: 10.1007/s00439-016-1640-3 (PMC4796337; doi:10.1007/s00439-016-1640-3)
Supplement: Supplementary file 1 — Supplementary material 1 (PDF 1617 kb) [file 439_2016_1640_MOESM1_ESM.pdf]

Online Resource 1

Quantifying the legacy of the Chinese Neolithic on the maternal genetic heritage of Taiwan and Island Southeast Asia

Andreia Brandão[1,2,3,4], Khen Khong Eng[5,6], Teresa Rito[1,7,8], Bruno Cavadas[1,2], David Bulbeck[9], Francesca Gandini[3], Maria Pala[3], Maru Mormina[5,10], Bob Hudson[11], Joyce White[12], Tsang-Ming Ko[13], Mokhtar Saidin[6], Zainuddin Zafarina[14,15], Stephen Oppenheimer[16], Martin B Richards[3,5,+], Luísa Pereira[1,2,17,+], Pedro Soares[1,2,5,18,+]

[1]IPATIMUP (Institute of Molecular Pathology and Immunology of the University of Porto), Rua Dr. Roberto Frias s/n, 4200-465 Porto, Portugal

[2]i3S (Instituto de Investigação e Inovação em Saúde, Universidade do Porto), 4200 Porto, Portugal.

[3]Department of Biological Sciences, School of Applied Sciences, University of Huddersfield, Queensgate, Huddersfield, HD1 3DH, United Kingdom

[4]ICBAS (Instituto Ciências Biomédicas Abel Salazar), Universidade do Porto, Rua de Jorge Viterbo Ferreira n.º 228, 4050-313 Porto, Portugal.

[5]Faculty of Biological Sciences, University of Leeds, LS2 9JT Leeds, United Kingdom

[6]Centre for Global Archaeological Research, Universiti Sains Malaysia, 11800 USM Penang, Malaysia

[7]Life and Health Sciences Research Institute (ICVS), School of Health Sciences, University of Minho, Braga, Portugal

[8]ICVS/3B's - PT Government Associate Laboratory, Braga/Guimarães, Portugal

[9]Department of Archaeology and Natural History, College of Asia and the Pacific, The Australian National University, Acton ACT 2601, Canberra, Australia

[10]Department of Applied Social Studies, University of Winchester, Sparkford Road, Winchester SO22 4NR, United Kingdom

[11]Archaeology Department, University of Sydney, New South Wales 2006, Australia

[12]Dept. of Anthropology, University of Pennsylvania Museum, 3260 South St. Philadelphia, United States of America.

[13]Department of Obstetrics and Gynecology, National Taiwan University, Roosevelt Rd., Taipei 10617, Taiwan

[14]Malaysian Institute of Pharmaceuticals and Nutraceuticals Malaysia, National Institutes of Biotechnology Malaysia, Penang, Malaysia

[15]Human Identification Unit, School of Health Sciences, Health Campus, Universiti Sains Malaysia, Kelantan, Malaysia.

[16]Institute of Human Sciences, School of Anthropology, University of Oxford, The Pauling Centre, 58a Banbury Road, Oxford OX2 6QS, United Kingdom

[17]Faculty of Medicine, University of Porto, Al. Prof. Hernâni Monteiro, 4200 - 319 Porto, Portugal

[18]CBMA (Centre of Molecular and Environmental Biology), Department of Biology, University of Minho, Campus de Gualtar, 4710-057 Braga, Portugal

[+]These authors contributed equally to this work

Correspondence should be addressed to:

Professor Martin B. Richards;

Department of Biological Sciences, School of Applied Sciences, University of Huddersfield, Queensgate, Huddersfield, HD1 3DH, United Kingdom

email: m.b.richards@hud.ac.uk

Telephone number: +44 1484 471676

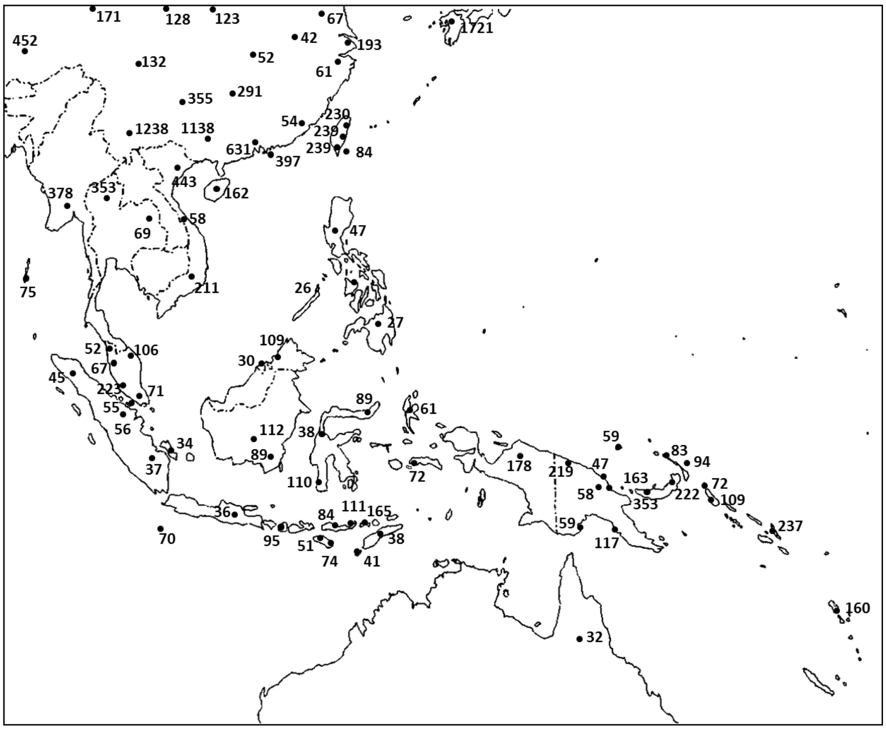

Figure S1. Map showing the geographic distribution and the sample sizes for the dataset used in the Surfer analyses.

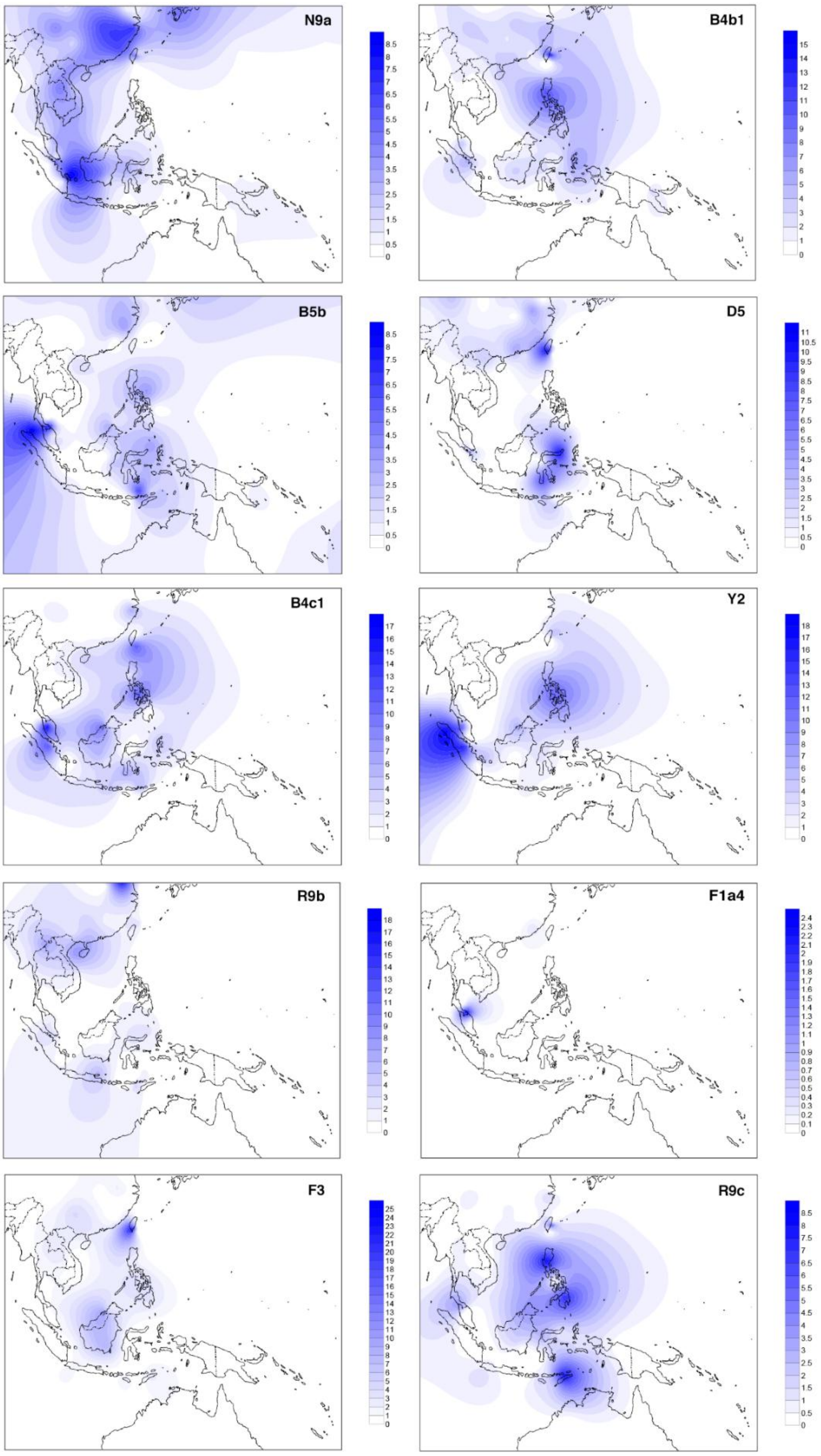

Figure S2. Frequency distribution maps for mtDNA haplogroups examined in this study, based on HVS-I data. Maps created using Surfer.

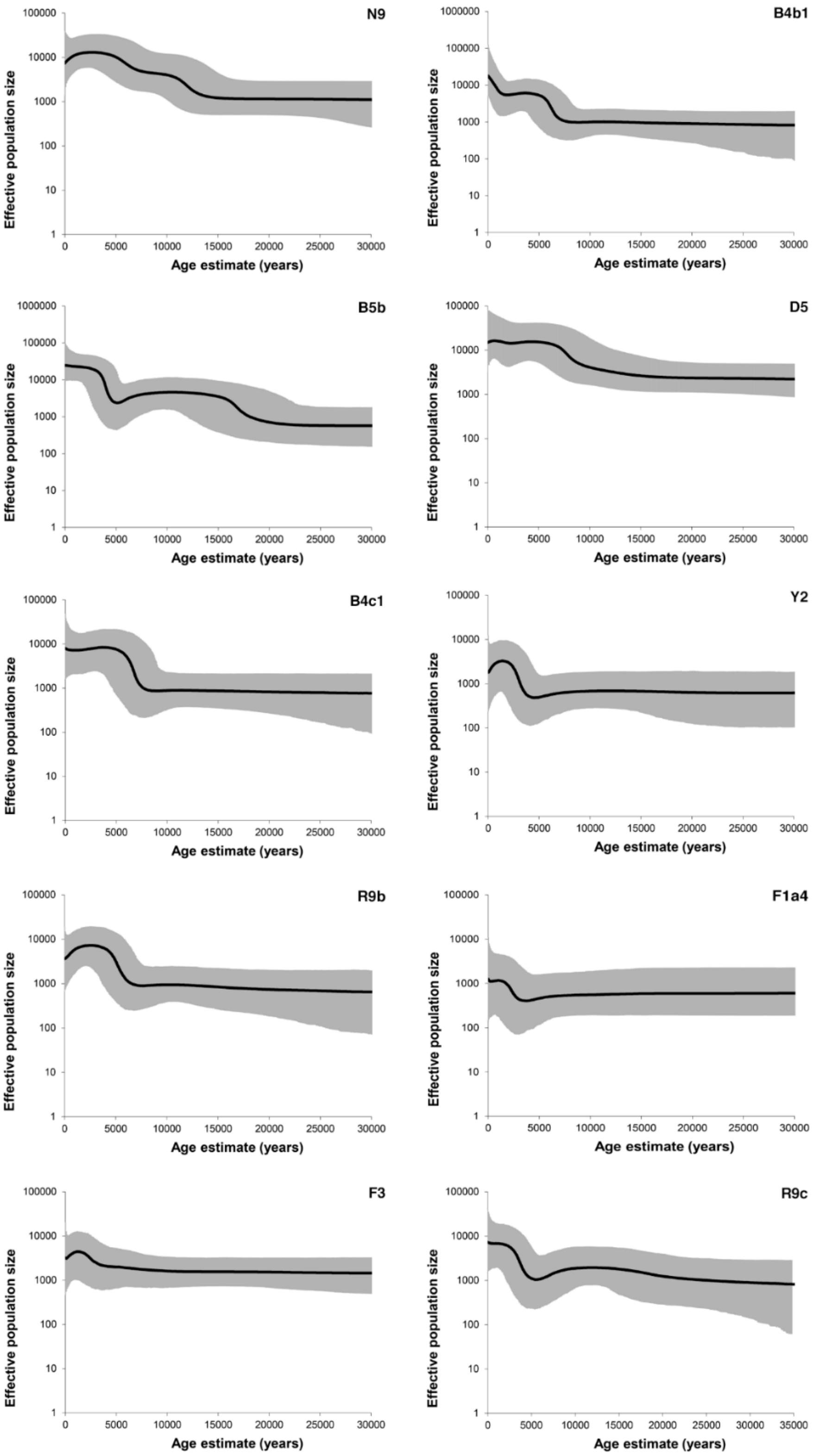

Figure S3. Bayesian skyline plots for mtDNA haplogroups examined in this study, assuming a generation time of 25 years. The black lines represent the posterior median of the effective population size through time, and the grey regions represent the 95% confidence interval.

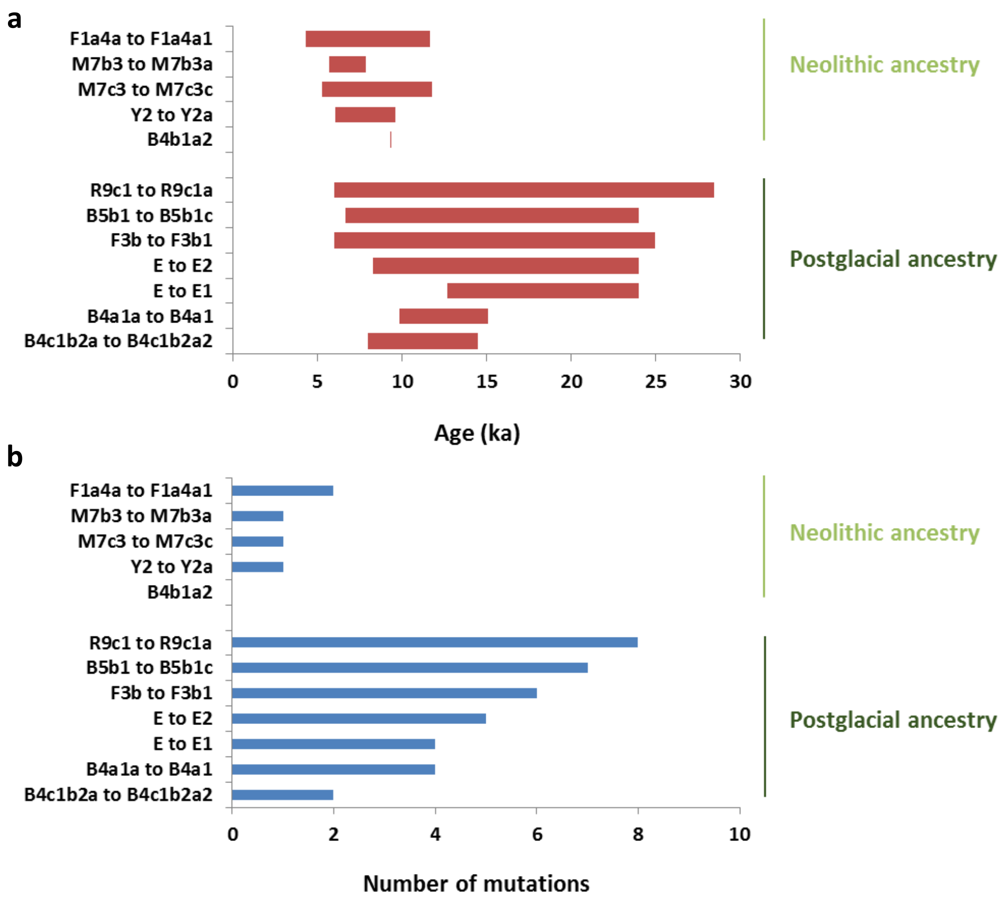

Figure S4. Phylogeographic patterns in ISEA. (a) ML ages of key mtDNA clades in ISEA and their ancestral nodes. (b) Number of mutations between key mtDNA clades in ISEA and their ancestral nodes.

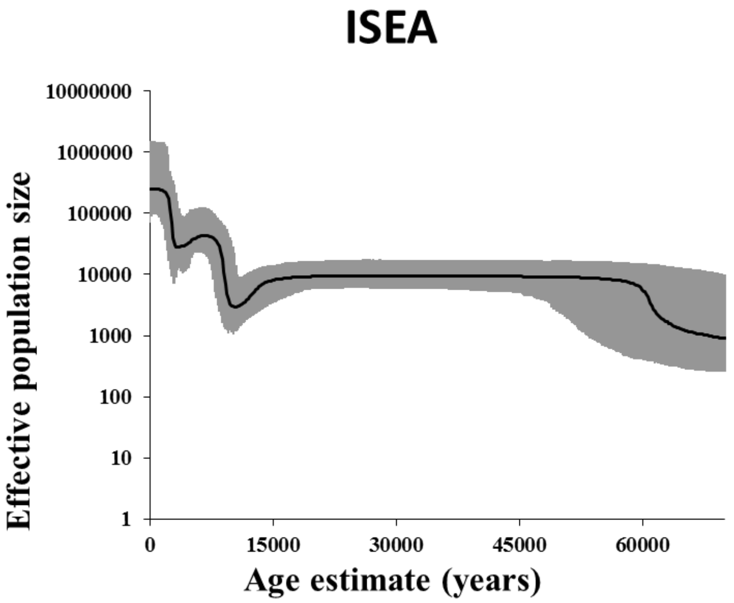

Figure S5. Bayesian skyline plots for ISEA, with the whole-mtDNA data set available, assuming a generation of 25 years. The black line represents the posterior effective population size through time, and the grey regions represents the 95% confidence interval.

Table S1. List of the 114 whole-mtDNA genomes sequenced and characterized in this study and corresponding geographic region.

Sample ID	Location	Haplogroup
BAN13	Indonesia, South Kalimantan	B4b1a2
BJ135	Malaysia, Perak, Banjar Malay	B4b1a2
KA34	Taiwan, Ami	B4b1a2
KB103	Micronesia, Kiribati	B4b1a2
KB67	Taiwan, Bunun	B4b1a2
KB71	Taiwan, Bunun	B4b1a2
KT55	Taiwan, Tsou	B4b1a2
NAU2	Micronesia, Nauru	B4b1a2
DOX2185	Vietnam, Tay Nung	B4b1a2a
DOX3064	Vietnam, Tay Nung	B4b1a2a
DOX4368	Vietnam, Kinh	B4c1b
DOX4329	Vietnam, Kinh	B4c1b2
AMB02	Indonesia, Ambon	B4c1b2a2
BG094	Malaysia, Johor, Bugis Malay	B4c1b2a2
BJ133	Malaysia, Perak, Banjar Malay	B4c1b2a2
BRU40	Brunei	B4c1b2a2
BW62	Malaysia, Jawa Malay	B4c1b2a2
KK94	Malaysia, Sabah	B4c1b2a2
KY32	Taiwan, Yami	B4c1b2a2
MB34	Malaysia, Kelantan Malay	B4c1b2a2
MI28	Malaysia, Negeri Sembilan, Minangkabau Malay	B4c1b2a2
MI30	Malaysia, Negeri Sembilan, Minangkabau Malay	B4c1b2a2
MI50	Malaysia, Negeri Sembilan, Minangkabau Malay	B4c1b2a2
MI61	Malaysia, Negeri Sembilan, Minangkabau Malay	B4c1b2a2
RP02	Malaysia, Kelantan Malay	B4c1b2a2
RW179	Malaysia, Perak, Rawa Malay	B4c1b2a2
DOX3048	Vietnam, Tay Nung	B4c1b2c
RW161	Malaysia, Rawa Malay	B5b1c
JW83	Malaysia, Jawa Malay	B5b1c
8A	Malaysia, Batek Malay	B5b1c
KP01	Taiwan, Paiwan	D5
WA4	Myanmar (Burma), Pakokku, Burmese	D5a2a1+!16172
DKK4149	Vietnam, Kinh	D5b
DOX2196	Vietnam, Tay Nung	D5b
DOX6681	Vietnam, Tay Nung	D5b
KP28	Taiwan, Paiwan	D5b
KP67	Taiwan, Paiwan	D5b
PAI05	Taiwan, Paiwan	D5b

AMI01	Taiwan, Ami	D5b1c1
KA53	Taiwan, Ami	D5b1c1
MND24	Indonesia, Manado	D5b1c1
DKX4098	Vietnam, Kinh	D5c+16311
MB39	Malaysia, Kelantan Mala	F1a4a
DHX4312	Vietnam, Kinh	F3a
VNM293	Vietnam	F3a
MB33	Malaysia, Kelantan Mala	F3a+207
BRU36	Brunei	F3a1
DOX701	Vietnam, Tay Nung	F3a1
DOX708	Vietnam, Tay Nung	F3a1
KDH05	Vietnam, Tay Nung	F3a1
LAO5-03	Laos, Hmong	F3a1
LAO5-05	Laos, Hmong	F3a1
LAO5-11	Laos, Hmong	F3a1
ALO162	Indonesia, Alor	F3b1
BAN02	Indonesia, South Kalimantan	F3b1
BRU58	Brunei	F3b1
FIL56	Philippines, Surigaonon	F3b1
KK43	Malaysia, Sabah	F3b1
KP42	Taiwan, Paiwan	F3b1
KP50	Taiwan, Paiwan	F3b1
KP57	Taiwan, Paiwan	F3b1
KP70	Taiwan, Paiwan	F3b1
MC01	Malaysia, Kelantan Malay	F3b1
PAI02	Taiwan, Paiwan	F3b1
PAI17	Taiwan, Paiwan	F3b1
PRY127	Indonesia, Palangkaraya	F3b1
C72	China	N9a1
DOX2193	Vietnam, Tay Nung	N9a10
DOX2221	Vietnam, Tay Nung	N9a10
LAO1-08	Laos, Hmong	N9a10
AMI16	Taiwan, Ami	N9a10a
KA40	Taiwan, Ami	N9a10a
KT11	Taiwan, Tsou	N9a10a
C84	China	N9a1'3
PAI10	Taiwan, Paiwan	N9a3
DKK4276	Vietnam, Kinh	N9a6
DKK4471	Vietnam, Kinh	N9a6
DOX2000	Vietnam, Tay Nung	N9a6
DOX4107	Vietnam, Stieng	N9a6
MI41	Malaysia, Negeri Sembilan, Minangkabau Malay	N9a6
RP32	Malaysia, Kelantan Malay	N9a6
136B	Malaysia, Temuan	N9a6a

147A	Malaysia, Temuan	N9a6a
KS31	Malaysia, Kensiu	N9a6a
PRY51	Indonesia, Palangkaraya	N9a6a
PRY71	Indonesia, Palangkaraya	N9a6a
WA1	Myanmar (Burma), Pakokku, Burmese	R9b1
100A	Malaysia, Semelai	R9b1a1a
101A	Malaysia, Semelai	R9b1a1a
108A	Malaysia, Semelai	R9b1a1a
KT03	Malaysia, Jahai, Semang	R9b1a1a
KT38	Malaysia, Kintak	R9b1a1a
RP15	Malaysia, Kelantan Malay	R9b1a1a
WT7	Myanmar (Burma), Pakokku, Burmese	R9b1b
MB12	Malaysia, Kelantan Malay	R9b2
DKK4295	Vietnam, Kinh	R9c1
THAI75	Thailand	R9c1
VNM221	Vietnam	R9c1
ALO153	Indonesia, Alor	R9c1a
ALO26	Indonesia, Alor	R9c1a
KP58	Taiwan, Paiwan	R9c1a
PRY76	Indonesia, Palangkaraya	R9c1a
BRU51	Brunei	Y2a
BRU60	Brunei	Y2a
KT17	Taiwan, Tsou	Y2a
AC02	Malaysia, Kedah, Acheh Malay	Y2a1
BGK24	Indonesia, Sumatra	Y2a1
BJ146	Malaysia, Perak, Banjar	Y2a1
FIL01	Philippines, Surigaonon	Y2a1
FIL08	Philippines, Surigaonon	Y2a1
KK13	Malaysia, Sabah	Y2a1
MED105	Indonesia, Sumatra	Y2a1
MED142	Indonesia, Sumatra	Y2a1
MND64	Indonesia, Sulawesi	Y2a1

Table S2. List of the 829 published whole mitochondrial genomes used for the phylogeographic analysis with the corresponding origin and haplogroup affiliation.

Haplogroup	Sample ID	Location	Reference
B4b1	HG00446, HG01798, HG02402, NA18110, NA18123, NA18627, NA18689, NA18103, NA18744,	China	(The 1000 Genomes Project Consortium 2012)
	HG02064, HG01866, HG02069,	Vietnam	(The 1000 Genomes Project Consortium 2012)
	AP010733, AP010735, AP010740, AP010671	Japan	(Bilal et al. 2008)
	KC993915, KC993921, KC993928, KC993932, KC993933, KC993936, KC993939, KC993941, KC993943, KC993945, KC993946, KC993949, KC993953, KC993971, KC994031, KC994039, KC994043, KC994127, KC994135, KC994140, KC994152, KC994146	Philippines	(Delfin et al. 2014)
	JN857022, JN857011, JN857014, JN857026, JN857036, JN857037, JN857043, JN857044, JN857045, JN857059	Russia	(Derenko et al. 2012)
	KF148375	Russia	(Duggan et al. 2013)
	GU733803, GU733813, GU733815, GU733818, GU733811, GU733814, GU733825, GU733826	Philippines, Surigaonon	(Gunnarsdóttir et al. 2011a)
	GU733790	Philippines, Manobo	(Gunnarsdóttir et al. 2011a)
	GU733738, GU733720, GU733724, GU733744, GU733747, GU733748, GU733750	Philippines, Mamanwa; negrito group	(Gunnarsdóttir et al. 2011a)
	EU597553	China	(Hartmann et al. 2009)
	KF540519, KF540523, KF540533, KF540542, KF540573, KF540607, KF540608, KF540610, KF540616, KF540617, KF540621, KF540627, KF540628, KF540629, KF540635, KF540640, KF540646, KF540651, KF540652, KF540653, KF540655, KF541023, KF541024, KF541028, KF541037, KF541041, KF540815, KF540644, KF540675, KF540810, KF540811, KF540838, KF540671	Taiwan	(Ko et al. 2014)
	AY255170	China	(Kong et al. 2003)
	DQ272119	China	(Kong et al. 2006)
	HQ873567	Vietnam	(Soares et al. 2011)
	AY519494	Russia	(Starikovskaya et al. 2005)
	AP008325, AP008335, AP008793, AP008898, AP008900, AP008904,	Japan	(Tanaka et al. 2004)

	AP008654, AP008665, AP008848, AP008917, AP008856, AP008682		
	NA18949, NA18984, NA19077	Japan	(The 1000 Genomes Project Consortium 2012)
B4c1	HG00596, HG00623, HG00704, NA18156, NA17979, NA18111, NA18552, NA18778, NA19574, NA19065	China	(The 1000 Genomes Project Consortium 2012)
	NA18982, NA19088	Japan	(The 1000 Genomes Project Consortium 2012)
	AP010679, AP010769, AP010696, AP010704, AP010718, AP010755	Japan	(Bilal et al. 2008)
	KC521454, KC993908, KC993927, KC994072, KC994082, KC994083, KC994085, KC994144	Philippines	(Delfin et al. 2014)
	JN857009, JN857019, JN857021, JN857025, JN857031, JN857035	Russia	(Derenko et al. 2012)
	GU733770, GU733773, GU733783, GU733785, GU733786, GU733795, GU733796, GU733800	Philippines	(Gunnarsdóttir et al. 2011a)
	HM596646, HM596691, HM596697	Indonesia, Sumatra	(Gunnarsdóttir et al. 2011b)
	KF540737, KF540666, KF540964, KF540965, KF540969, KF540976, KF540978, KF540979, KF540984, KF540985, KF540986, KF540988, KF540990, KF540992, KF540993, KF541002, KF541003, KF541004, KF540661, KF540719	Taiwan	(Ko et al. 2014)
	AY255149	China	(Kong et al. 2003)
	HM238198, HM238200	Philippines, Ivatan	(Loo et al. 2011)
	HM238214, HM238215	Taiwan, Orchid Islands, Yami	(Loo et al. 2011)
	AP008669, AP008298, AP008688, AP008281, AP008899, AP008831, AP008920, AP008269	Japan	(Tanaka et al. 2004)
B5b	JX024567	China	(Bi et al. 2012)
	AP010771, AP010686, AP010689, AP010761	Japan	(Bilal et al. 2008)
	KC994086, KC994070, KC994000, KC993920, KC993904, KC993903, KC994143, KC994112, KC994111, KC994093, KC994054, KC994036, KC994004, KC994138, KC994128, KC994124, KC994117, KC994095, KC994094, KC994092, KC994058, KC994048, KC994046, KC994042, KC994028, KC993926	Philippines	(Delfin et al. 2014)
	JN857039, JN857016, JN857015	Russia	(Derenko et al. 2012)
	GU733794, GU733765	Philippines, Manobo	(Gunnarsdóttir et al. 2011a)
	GU733731	Philippines, Mamanwa; negrito group	(Gunnarsdóttir et al. 2011a)
	AP009425	Unknown	(Kazuno et al. 2005)

	KF540694, KF540850, KF540720	Taiwan	(Ko et al. 2014)
	AY255179	China	(Kong et al. 2003)
	HM238201	Philippines, Ivatan	(Loo et al. 2011)
	AB055387	Japan	(Shin et al. 2000)
	AY519489	Russia	(Starikovskaya et al. 2005)
	GU903056	Russia	(Sukernik et al. 2010)
	GQ119020, GQ119041	Philippines	(Tabbada et al. 2010)
	AP008284, AP008827, AP008687, AP008631, AP008852, AP008802, AP008751, AP008288, AP008273, AP008875, AP008518, AP008847, AP008737, AP008546, AP008488, AP008323, AP008846, AP008371, AP008910, AP008580, AP008491, AP008403, AP008584, AP008465, AP008566	Japan	(Tanaka et al. 2004)
	AP011050, AP011059	Japan	(Ueno et al. 2009)
	KC505068	Cambodia	(Zhang et al. 2013)
	HG00610, NA18704, NA18758, HG00690, HG00404	China	(The 1000 Genomes Project Consortium 2012)
	NA18989, NA19009, NA18985, NA18961	Japan	(The 1000 Genomes Project Consortium 2012)
	GU377081	China	(Zou et al. 2010)
D5	HG02190, HG00595, HG00475, HG00684, HG00657, HG00702, HG02152, HG00580, NA18531, NA18533, NA18630, NA18757, HG00628, NA18623, NA18640, NA19054, NA18558, NA17962, NA19002, NA18753, NA18576, NA18767, NA18106, NA18605, NA19074, NA18113	China	(The 1000 Genomes Project Consortium 2012)
	NA19006	Japan	(The 1000 Genomes Project Consortium 2012)
	HG01852	Vietnam	(The 1000 Genomes Project Consortium 2012)
	JQ702264	Finland, Saami	(Behar et al. 2012)
	JQ702153	Unknown	(Behar et al. 2012)
	FJ383197, FJ383201, FJ383204, FJ383208, FJ383180, FJ383190, FJ383195, FJ383206, FJ383209	India	(Kumar et al. 2009)
	KC994014, KC994015, KC994019	Philippines	(Delfin et al. 2014)
	FJ951453	Polish	(Derenko et al. 2010)
	FJ951589	Korean	(Derenko et al. 2010)
	FJ951467, FJ951466, FJ951479,	Russia	(Derenko et al.

	FJ951465, FJ951513, FJ951567, FJ951615		2010)
	FJ951513	North Asia, Mongolia	(Derenko et al. 2010)
	FJ951567	North Asia, Evenk	(Derenko et al. 2010)
	KF148068, KF148070, KF148073, KF148074, KF148078, KF148081, KF148095, KF148097, KF148098, KF148120, KF148128, KF148130, KF148181, KF148197, KF148202, KF148258, KF148328, KF148330, KF148385, KF148386, KF148389, KF148393, KF148398, KF148399, KF148400, KF148404, KF148410, KF148428, KF148431, KF148438, KF148444, KF148451, KF148452, KF148462, KF148465, KF148487, KF148547, KF148562, KF148563, KF148588,	Russia	(Duggan et al. 2013)
	EU428195	Norway, Saami	Family Tree DNA
	EU597530	Russia	(Hartmann et al. 2009)
	EU007869	North Asia, Kazakh	(Ingman and Gyllensten 2007)
	EU007893	North Asia, Mongolia	(Ingman and Gyllensten 2007)
	EU007881	North Asia, Shortci	(Ingman and Gyllensten 2007)
	FJ748752, FJ748730	Tibet	(Ji et al. 2012)
	KF540674, KF540717, KF540926, KF540632, KF540723, KF540738, KF540751, KF540755, KF540757, KF540758, KF540786, KF540800, KF540841, KF540882, KF540890, KF540891, KF540893, KF540895, KF540896, KF540900, KF540902, KF540904, KF540906, KF540909, KF540910, KF540914, KF540915, KF540920, KF540922, KF540923, KF540924, KF540929, KF540930, KF540933, KF540934, KF540935, KF540938, KF540939, KF540746, KF540508, KF540520, KF540529, KF540541, KF540704, KF540856, KF540733,	Taiwan	(Ko et al. 2014)
	AY255162, AY255169	China	(Kong et al. 2003)
	FJ147320	Russia	(Sukernik et al. 2012)
	JX266269	Polish, Podhale	(Mielnik-Sikorska et al. 2013)
	HM036558	India	Sharma et al. (unpublished)
	AY570524, AY570525	Russia	(Starikovskaya et al. 2005)
	GQ119025	Philippines	(Tabbada et al. 2010)

	AP008674	Japan	(Tanaka et al. 2004)
	EU482378, EU482309, EU482311, EU482327, EU482373, EU482335, EU482336	Russia	(Volodko et al. 2008)
	JF271013, JF271014	China	Zhu,M (unpublished)
F1a4	HG02029	Vietnam	(The 1000 Genomes Project Consortium 2012)
	KC993906, KC993931, KC993975, KC993976, KC993978, KC993986, KC994007, KC994022, KC994108	Philippines	(Delfin et al. 2014)
	HM804484	Philippines	Family Tree DNA
	GU733782	Philippines, Manobo	(Gunnarsdóttir et al. 2011a)
	HM596654	Indonesia, Sumatra	(Gunnarsdóttir et al. 2011b)
	KF540547, KF540612, KF540808, KF540818, KF541016, KF541017, KF541030, KF541031, KF541055	Taiwan	(Ko et al. 2014)
	HM238204	Philippines, Ivatan	(Loo et al. 2011)
	HQ700871	Guam, Mariana Islands	(Reiff et al. 2011)
	NA18132	China	(The 1000 Genomes Project Consortium 2012)
F3	HG01867	Vietnam	(The 1000 Genomes Project Consortium 2012)
	HG01797, HG02360, HG02363, HG02375, HG02388, NA18122, NA18759	China	(The 1000 Genomes Project Consortium 2012)
	AY972053	China	(Bandelt et al. 2005)
	KC993991, KC994009, KC994010, KC994023, KC994030, KC994056, KC994118, KC994125, KC994139, KC994154	Philippines	(Delfin et al. 2014)
	HM596715	Indonesia, Sumatra	(Gunnarsdóttir et al. 2011b)
	FJ748712, FJ748722	Tibet	(Ji et al. 2012)
	KF540698, KF540550, KF540611, KF540624, KF540633, KF540638, KF540752, KF540768, KF540778, KF540794, KF540795, KF540799, KF540816, KF540848, KF540853, KF540857, KF540864, KF540865, KF540866, KF540868, KF540871, KF540873, KF540878, KF540881, KF540889, KF540899, KF540903, KF540908, KF540916, KF540919, KF540921, KF540927, KF540928, KF540931, KF540937, KF541035, KF541051	Taiwan	(Ko et al. 2014)
	AY255167	China	(Kong et al. 2003)
	JF739538	Philippines, Palawan Island	(Scholes et al. 2011)

	GQ119011	Indonesia	(Tabbada et al. 2010)
	GQ119009, GQ119014, GQ119015	Taiwan	(Tabbada et al. 2010)
N9	AP012369, AP012372, AP012373, AP012384, AP012421, AP012393, AP012395, AP012396, AP012397, AP012398, AP012399, AP012401, AP012402, AP012403, AP012404, AP012408, AP012410, AP012411, AP012412, AP012413	Malaysia	(Jinam et al. 2012)
	HG00479, HG00500, HG00592, JN857052, HG00406, HG00620, HG00556, HG00577, HG00584, NA18740, HG00531, HG00422, NA18747, NA18748	China	(The 1000 Genomes Project Consortium 2012)
	HG02521, HG01855, HG02040, HG02032	Vietnam	(The 1000 Genomes Project Consortium 2012)
	AP010744	Japan	(Bilal et al. 2008)
	JN857042, JN857023, JN857038, JN857053, JN857057, JN857027,	Russia	(Derenko et al. 2012)
	HM589048	China	Family Tree DNA
	HM596703	Indonesia, Sumatra	(Gunnarsdóttir et al. 2011b)
	FJ748719	Tibet	(Ji et al. 2012)
	KF540662, KF540722, KF540679, KF540684, KF540744	Taiwan	(Ko et al. 2014)
	DQ834255, DQ834258	Vietnam	Phan et al. (unpublished)
	AP008261, AP008726, AP008608, AP008714	Japan	(Tanaka et al. 2004)
	HM776708	Russia	(Sukernik et al. 2012)
R9b	HG02382, HG02410, HG01796, HG00663, NA18779, NA18781	China	(The 1000 Genomes Project Consortium 2012)
	HG01874, HG02017, HG02087, HG02035, HG02061, HG02020	Vietnam	(The 1000 Genomes Project Consortium 2012)
	GU733737	Philippines, Mamanwa; negrito group	(Gunnarsdóttir et al. 2011a)
	DQ981471, DQ981469, DQ981470, DQ981474, DQ981475,	Vietnam	(Hill et al. 2006)
	DQ981473	Thailand	(Hill et al. 2006)
	DQ981472	Malaysia, Orang sli	(Hill et al. 2006)
	DQ981465	Indonesia, Java	(Hill et al. 2006)
	DQ981466	Indonesia, Sumatra, Palembang	(Hill et al. 2006)
	DQ981467	Indonesia, Sumatra, Padang	(Hill et al. 2006)
	DQ981468	Indonesia, Sulawesi, Manado	(Hill et al. 2006)
	KF540682, KF540702, KF540748, KF541026, KF541027	Taiwan	(Ko et al. 2014)
	AY255152	China	(Kong et al. 2003)

	AY963579	Malaysia, Semelai	(Macaulay et al. 2005)
	FJ147308	Russia	(Sukernik et al. 2012)
	GU810062	Thailand	Pradutkanchana et al. (unpublished)
	EF114273, EF114274, EF114275	China	(Wang et al. 2007)
	JQ411477	China	Zhang et al. (unpublished)
R9c	HG00534, HG00445, HG00407	China	(The 1000 Genomes Project Consortium 2012)
	KC994001, KC994055, KC994057, KC994066, KC994068, KC994078, KC994160	Philippines	(Delfin et al. 2014)
	JN857032	North Asia, Mongolia	(Derenko et al. 2012)
	GU733759	Philippines, Manobo	(Gunnarsdóttir et al. 2011a)
	KF540813, KF540625, KF540804, KF540806, KF540831, KF540842, KF540867, KF540898, KF540911, KF541010, KF541011, KF541013, KF541021, KF541022, KF541033, KF541039, KF541043, KF541045, KF541049, KF541050	Taiwan	(Ko et al. 2014)
	JF739535, JF739539	Philippines, Palawan Island	(Scholes et al. 2011)
	JX289135	Myanmar (Burma)	(Summerer et al. 2014)
	GQ119007	Taiwan	(Tabbada et al. 2010)
	GQ119010	Indonesia	(Tabbada et al. 2010)
Y	HG02026	Vietnam	(The 1000 Genomes Project Consortium 2012)
	KC994149, KC993979, KC993980, KC993982, KC993988, KC993992, KC994003, KC994035, KC994040, KC994060, KC994067, KC994129, KC994130, KC994131, KC994134, KC994137	Philippines	(Delfin et al. 2014)
	EF153813, EF153825, EF153812, EF153798	Russia	(Derenko et al. 2007)
	KF148108, KF148113, KF148129, KF148143, KF148335, KF148337, KF148339, KF148340, KF148341, KF148342, KF148343, KF148344, KF148345, KF148346, KF148347, KF148348, KF148349, KF148352, KF148353, KF148354, KF148359, KF148361, KF148362, KF148363, KF148365, KF148368, KF148370, KF148371, KF148372, KF148373, KF148486, KF148507, KF148508, KF148513, KF148525	Russia	(Duggan et al. 2013)
	GU733733	Philippines, Mamanwa;	(Gunnarsdóttir et

		negrito group	al. 2011a)
	GU733823	Philippines, Surigaonon	(Gunnarsdóttir et al. 2011a)
	GU733768, GU733787, GU733798	Philippines, Manobo	(Gunnarsdóttir et al. 2011a)
	HM596648, HM596672, HM596675	Indonesia, Sumatra	(Gunnarsdóttir et al. 2011b)
	EU007848, EU007853, EU007854, EU007855, EU007892	North Asia, Mongolia	(Ingman and Gyllensten 2007)
	AP009439	Japan	(Kazuno et al. 2005)
	KF540727, KF540560, KF540577, KF540941	Taiwan	(Ko et al. 2014)
	DQ272121	China	(Kong et al. 2006)
	GU123044	Russia	(Malyarchuk et al. 2010)
	AY195792	Asia	(Mishmar et al. 2003)
	AY255138	Asia	(Kong et al. 2003)
	GQ119016, GQ119013, GQ119019, GQ119032	Taiwan	(Tabbada et al. 2010)
	AP008723, AP008764	Japan	(Tanaka et al. 2004)
	NA18134	China	(The 1000 Genomes Project Consortium 2012)
	NA18974	Japan	(The 1000 Genomes Project Consortium 2012)

Table S3. Age estimates using rho (ρ) and ML for haplogroups B4b1, B4c1, B5b, D5, F1a4, F3, N9a, R9b, R9c and Y2, and its major subclades. Ages and 95% confidence intervals (CI) in thousands of years.

Nodes	N	PAML		Rho			
				Total		Synonymous	
		Age	CI	Age	CI	Age	CI
B4	226	46,500	[33,900–59,600]	39,500	[27,000–52,500]	45,100	[23,000–67,600]
B4b	130	28,400	[18,500–38,700]	25,500	[14,000–37,700]	32,600	[9,500–55,600]
B4b1	129	25,100	[17,000–33,600]	22,600	[12,600–33,200]	32,600	[9,400–55,900]
B4b1+150	2	11,000	[1,500–20,900]	10,600	[3,200–18,400]	15,800	[300–31,200]
B4b1a	119	19,900	[10,700–29,400]	17,000	[9,700–24,700]	17,800	[5,400–30,300]
B4b1a+207	117	17,700	[10,200–25,500]	14,200	[9,100–19,500]	17,700	[5,100–30,400]
B4b1a1	16	4,400	[2,300–6,700]	4,600	[2,100–7,000]	4,900	[1,900–8,000]
B4b1a1a	6	2,600	[600–4,600]	2,600	[500–4,700]	1,300	[0–3,900]
B4b1a2	89	9,300	[6,800–11,800]	8,700	[6,400–11,000]	9,100	[5,300–12,900]
B4b1a2+1313	4	1,200	[0–3,600]	900	[0–2,600]	0	[0–0]
B4b1a2+14783	5	600	[0–1,900]	500	[0–1,500]	0	[0–0]
B4b1a2+16365	16	3,600	[0–9,000]	3,700	[500–7,100]	8,900	[0–18,200]
B4b1a2+16465	3	3,400	[0–9,200]	2,600	[0–6,400]	2,600	[0–7,800]
B4b1a2+178	2	1,600	[0–10,800]	1,300	[0–3,800]	0	[0–0]
B4b1a2+204	2	4,800	[0–8,700]	7,900	[1,500–14,500]	0	[0–0]
B4b1a2+207	4	4,600	[0–10,300]	5,200	[400–10,200]	9,900	[0–22,700]
B4b1a2+709	9	4,100	[0–14,700]	3,700	[300–7,200]	7,900	[0–17,100]
B4b1a2a	9	7,900	[5,100–10,500]	12,400	[5,400–19,800]	14,00	[0–29,200]
B4b1a2a+150	2	5,400	[0–11,300]	5,200	[100–10,500]	4,000	[0–11,700]
B4b1a2a+15301	7	7,200	[4,100–10,300]	11,000	[4,000–18,300]	9,000	[0–20,700]
B4b1a2b	11	3,700	[0–7,600]	5,200	[1,300–9,300]	5,700	[0–13,400]
B4b1a2b+8659	5	1,900	[0–4,500]	5,200	[600–9,900]	3,200	[0–9,300]
B4b1a2c	6	1,500	[0–5,700]	1,700	[0–5,100]	5,300	[0–15,600]
B4b1a2c+11809	4	500	[0–4,100]	0	[0–0]	0	[0–0]
B4b1a2d	4	600	[0–1,700]	600	[0–1,900]	2,000	[0–5,800]
B4b1a3	12	9,500	[2,800–16,400]	11,100	[3,200–19,400]	11,200	[0–24,700]
B4b1a3a	10	3,200	[400–5,900]	3,400	[1,000–5,700]	4,000	[0–8,600]
B4b1a3a+4026	2	1,200	[0–3,400]	1,300	[0–3,800]	0	[0–0]
B4b1a3a+827	2	800	[0–2,300]	1,300	[0–3,800]	4,000	[0–11,700]
B4b1b'c	8	21,700	[13,400–30,300]	17,900	[8,900–27,400]	22,700	[2,900–42,600]

B4b1c	2	17,100	[9,300–25,300]	14,200	[5,800–22,900]	15,800	[0–32,000]
B4b1c+8343	2	12,300	[4,300–20,700]	9,200	[2,300–16,400]	3,900	[0–11,700]
B4b1c1	5	5,600	[2,200–9,100]	4,200	[1,300–7,100]	4,700	[0–10,100]
B4b'd'e'j	131	37,100	[25,400–49,300]	34,300	[18,900–50,700]	48,000	[16,500–79,600]
B4c	95	36,800	[23,500–50,800]	32,000	[18,300–46,500]	22,400	[8,800–36,100]
B4c1	94	32,000	[18,300–46,600]	29,200	[16,700–42,300]	22,500	[8,700–36,200]
B4c1a	17	12,400	[4,300–21,000]	9,800	[5,300–14,400]	10,200	[5,200–15,200]
B4c1a1	11	9,200	[5,400–13,200]	9,400	[5,700–13,100]	12,200	[5,200–19,200]
B4c1a1a	3	8,200	[4,500–12,000]	13,400	[6,000–21,000]	10,500	[0–23,100]
B4c1a1a1	2	7,400	[3,800–11,200]	16,200	[6,800–25,100]	3,900	[0–11,700]
B4c1a1b	2	7,400	[3,100–11,900]	9,200	[2,300–16,400]	15,800	[300–31,200]
B4c1a1c	2	2,400	[0–6,400]	2,600	[0–6,200]	3,900	[0–11,700]
B4c1a2	5	6,200	[2,000–10,700]	4,200	[1,200–7,100]	7,900	[1,000–14,800]
B4c1a'b	90	29,000	[15,000–43,600]	26,300	[14,700–38,500]	22,600	[8,300–36,900]
B4c1b	73	24,600	[16,600–32,800]	20,800	[10,800–31,400]	23,700	[6,400–40,900]
B4c1b1	3	5,200	[700–9,800]	6,700	[600–13,700]	10,500	[0–25,100]
B4c1b1a	2	900	[0–2,700]	1,300	[0–3,800]	0	[0–0]
B4c1b2	69	20,700	[13,700–27,800]	17,600	[8,700–27,000]	23,900	[5,600–42,100]
B4c1b2+195	5	17,400	[7,300–27,900]	13,400	[5,900–21,200]	17,300	[2,500–32,200]
B4c1b2a	56	14,500	[6,400–23,000]	11,200	[3,800–19,000]	15,500	[0–31,400]
B4c1b2a+15301	54	10,800	[800–21,300]	8,400	[2,900–14,200]	7,900	[2,100–13,700]
B4c1b2a2	53	8,000	[5,600–10,500]	5,800	[3,500–8,100]	7,700	[1,900–13,600]
B4c1b2a2+3221	4	2,200	[0–9,500]	2,600	[0–6,700]	0	[0–0]
B4c1b2a2+3666	2	3,600	[0–7,600]	2,600	[0–6,200]	0	[0–0]
B4c1b2a2+4226	3	5,500	[1,700–9,500]	5,200	[0–10,800]	13,100	[0–28,600]
B4c1b2a2+4937	19	300	[0–700]	200	[0–600]	800	[0–1,900]
B4c1b2a2+6383	2	1,700	[0–4,900]	2,600	[0–6,200]	3,900	[0–11,700]
B4c1b2a2+7598	4	1,600	[0–4,400]	1,300	[0–3,100]	0	[0–0]
B4c1b2c	8	15,000	[9,200–21,000]	15,800	[9,600–22,300]	15,800	[6,300–25,200]
B4c1b2c+10493	3	2,600	[0–6,300]	5,200	[400–10,200]	7,900	[0–19,400]
B4c1b2c+16129	3	14,100	[8,300–20,100]	20,000	[10,300–30,100]	18,400	[4,800–32,000]
B4c1c	4	14,500	[5,500–23,900]	9,900	[3,200–17,000]	3,900	[0–9,400]
B4c1c1	3	8,600	[2,500–15,000]	7,900	[2,700–13,300]	5,300	[0–12,500]
B5	90	44,700	[33,300–56,500]	65,600	[43,200–89,300]	66,800	[34,300–99,200]
B5b	89	29,800	[20,700–39,300]	34,300	[24,300–44,700]	43,500	[24,500–62,500]
B5b+204	62	28,700	[19,600–38,200]	29,800	[18,900–41,100]	48,800	[23,400–74,200]
B5b1	54	23,900	[13,900–34,300]	27,000	[15,800–38,800]	44,700	[19,200–70,300]
B5b1+204	2	18,400	[5,200–32,500]	10,600	[3,200–18,400]	15,800	[300–31,200]
B5b1a	15	19,000	[8,500–29,800]	20,500	[9,300–32,400]	12,600	[0–25,800]

B5b1a1	3	7,400	[1,500–13,600]	7,000	[2,100–12,000]	5,300	[0–12,500]
B5b1a2	12	4,900	[1,900–8,000]	4,600	[2,200–6,900]	6,600	[1,200–12,000]
B5b1a2a	3	2,200	[0–8,300]	2,600	[0–6,400]	5,300	[0–15,600]
B5b1a2a+6929	2	879	[0–7,500]	1,300	[0–3,800]	0	[0–0]
B5b1c	34	6,700	[4,000–9,400]	8,600	[3,500–13,800]	15,000	[1000–29,300]
B5b1c+1275	2	1,300	[0–3,900]	1,300	[0–3,800]	0	[0–0]
B5b1c+234	2	1,100	[0–4,300]	0	[0–0]	0	[0–0]
B5b1c1	20	4,100	[1,000–7,300]	5,200	[1,200–9,400]	7,500	[0–17,800]
B5b1c1+103	7	2,600	[0–6,300]	3,300	[500–6,200]	4,500	[0–9,900]
B5b1c1a	13	2,000	[300–3,800]	2,200	[600–3,800]	1,200	[0–3,600]
B5b1c1a+16497	2	816	[0–2,400]	1,300	[0–3,800]	0	[0–0]
B5b1c1a+9455	2	816	[0–2,300]	1,300	[0–3,800]	0	[0–0]
B5b2	27	21,900	[15,200–28,900]	22,900	[14,000–32,100]	15,500	[8,400–22,600]
B5b2a	22	19,700	[12,900–26,900]	19,700	[10,800–28,700]	11,500	[4,800–18,100]
B5b2a+5964	2	15,400	[7,000–24,200]	12,000	[4,000–20,300]	19,700	[2,400–37,000]
B5b2a1	19	17,000	[10,000–24,400]	14,600	[8,300–21,100]	9,500	[2,300–16,800]
B5b2a1a	8	4,800	[1,500–8,100]	4,300	[1,700–6,700]	3,900	[100–7,800]
B5b2a1a+195	2	1,500	[0–4,500]	1,300	[0–3,800]	3,900	[0–11,700]
B5b2a1b	10	9,600	[4,900–14,500]	8,700	[4,300–13,200]	4,700	[900–8,500]
B5b2a1b1	5	8,400	[3,800–13,200]	9,000	[2,800–15,400]	1,600	[0–4,700]
B5b2a1b1+8894	4	2,700	[0–7,100]	2,600	[0–6,700]	0	[0–0]
B5b2c	3	11,300	[5,500–17,300]	13,400	[6,000–21,000]	21,000	[4,700–37,300]
B5b2c+9299	2	7,700	[1,900–13,700]	7,900	[1,500–14,500]	19,700	[2,400–37,000]
B5b3	4	13,200	[4,400–22,400]	13,400	[6,600–20,400]	3,900	[0–9,400]
B5b3b	3	11,400	[2,400–21,000]	7,900	[2,700–13,600]	2,600	[0–7,800]
F	116	49,400	[39,600–59,500]	60,000	[40,800–80,100]	49,100	[24,000–74,300]
F1	28	27,200	[16,300–38,700]	28,000	[13,100–43,800]	27,600	[2,900–52,400]
F1a	27	20,400	[11,100–30,200]	21,900	[9,000–35,600]	19,900	[0–40,300]
F1a4	26	16,300	[7,000–26,000]	18,600	[6,500–31,300]	11,500	[0–26,100]
F1a4a	25	11,700	[3,000–21,000]	10,600	[2,600–19,000]	11,700	[0–26,800]
F1a4a1	23	4,300	[1,800–6,800]	5,200	[1,500–9,000]	3,900	[800–7,100]
F1a4a1a	15	3,300	[1,300–5,300]	3,500	[1,500–5,500]	3,700	[1,000–6,400]
F1a4a1a+152	4	700	[0–2,000]	600	[0–1,900]	0	[0–0]
F3	88	31,700	[21,500–42,300]	37,900	[22,900–53,700]	35,400	[12,400–58,400]
F3a	20	26,600	[16,500–37,200]	31,500	[18,700–45,000]	26,400	[6,000–46,800]
F3a+195	4	11,800	[0–31,800]	13,400	[5,000–22,200]	0	[0–0]
F3a1	16	16,600	[9,000–24,500]	15,600	[9,300–22,200]	13,300	[1,280–25,300]
F3a1+16093	3	11,600	[3,000–20,500]	11,500	[3,500–20,000]	13,100	[0–26,800]
F3a1+16093+5913	2	1,300	[0–9,600]	1,300	[0–3,900]	0	[0–0]

F3a1+204	3	2,000	[0–5,700]	1,800	[0–4,100]	0	[0–0]
F3a1+9854	10	16,600	[8,500–25,000]	15,000	[7,700–22,700]	7,100	[0–16,700]
F3a1+9854+4824	7	2,400	[100–4,900]	1,800	[0–3,400]	1,300	[0–3,900]
F3a1+9854+7151	3	8,600	[2,800–14,700]	10,700	[3,600–17,900]	2,600	[0–7,800]
F3b	68	25,200	[15,400–35,400]	28,900	[13,600–45,100]	27,800	[13,500–52,200]
F3b+2392	3	5,700	[0–14,300]	3,500	[0–7,700]	2,600	[0–7,800]
F3b1	65	12,400	[5,200–20,000]	12,000	[4,700–19,700]	12,100	[0–25,100]
F3b1a'c	52	8,200	[1,700–14,900]	7,000	[2,200–11,900]	3,500	[1,100–5,900]
F3b1a	15	4,600	[1,700–7,500]	4,600	[2,100–6,900]	3,700	[1,000–6,400]
F3b1b	11	6,900	[3,300–10,400]	5,000	[1,600–8,500]	7,300	[0–16,200]
N9	254	50,600	[37,100–64,600]	38,452	[27,700–49,600]	36,100	[20,700–51,400]
N9a	127	20,000	[14,500–25,500]	17,500	[13,000–22,100]	18,400	[10,900–25,900]
N9a1	14	6,500	[3,800–9,200]	6,400	[4,000–8,800]	6,200	[2,200–10,200]
N9a1+12007	2	3,800	[700–6,700]	2,600	[0–6,200]	7,900	[0–18,800]
N9a10	18	16,600	[11,000–22,400]	14,600	[8,800–20,600]	14,900	[4,600–25,200]
N9a10+16189	3	5,000	[0–10,600]	3,500	[100–6,900]	2,700	[0–7,800]
N9a10+16311	3	8,400	[1,600–15,600]	6,100	[1,600–10,800]	5,300	[0–12,500]
N9a10+9055	2	12,100	[5,200–19,300]	13,400	[5,000–22,200]	3,900	[0–11,700]
N9a10a	9	10,000	[4,700–15,400]	9,100	[4,100–14,200]	14,000	[1,600–26,400]
N9a10a1	6	6,300	[200–12,500]	5,200	[1,200–9,300]	6,600	[0–14,300]
N9a10a+9821	2	8,300	[3,300–13,400]	10,600	[3,200–18,400]	11,800	[0–25,200]
N9a1'3	25	15,700	[8,800–22,800]	15,200	[8,400–22,200]	19,200	[6,400–32,300]
N9a1a	2	4,900	[1,600–8,200]	6,600	[800–12,500]	0	[0–0]
N9a2	18	9,600	[3,700–15,700]	9,300	[4,200–14,400]	7,900	[1,900–13,800]
N9a2'4'5	32	17,200	[10,200–24,600]	13,600	[8,200–19,200]	16,300	[5,700–26,800]
N9a2'4'5+195	4	2,300	[0–6,00]	2,600	[0–5,700]	0	[0–0]
N9a2a	13	6,500	[0–10,000]	6,600	[3,400–9,700]	5,600	[0–11,500]
N9a2a1	3	3,000	[0–6,600]	3,500	[100–6,900]	2,600	[0–7,800]
N9a2a2	4	2,300	[0–4,800]	2,000	[0–4,200]	2,000	[0–5,800]
N9a2a3	2	3,700	[73–7,400]	5,200	[100–10,500]	0	[0–0]
N9a2c	3	2,600	[0–7,800]	2,600	[0–5,600]	2,600	[0–7,800]
N9a3	10	8,800	[4,700–13,000]	7,900	[4,500–11,400]	11,800	[4,400–19,200]
N9a3+195	2	3,600	[0–9,500]	2,600	[0–6,200]	3,900	[0–11,700]
N9a3+4913	2	1,600	[0–4,600]	1,300	[0–3,800]	0	[0–0]
N9a4	9	5,300	[500–10,200]	4,000	[500–7,700]	9,600	[0–20,100]
N9a4a	5	1,500	[0–3,500]	1,600	[0–3,300]	1,600	[0–4,700]
N9a4b+9156	3	3,200	[0–7,900]	1,700	[0–4,200]	2,600	[0–7,800]
N9a6	45	14,800	[9,900–19,800]	12,700	[7,100–18,400]	9,600	[2,900–16,400]
N9a6+15080	2	10,200	[1,200–19,800]	7,900	[1,500–14,500]	11,800	[0–25,200]

N9a6+16292	3	13,600	[8,200–19,200]	21,000	[11,400–30,900]	21,000	[6,500–35,600]
N9a6+3849	3	10,600	[4,000–17,400]	8,800	[2,800–15,000]	7,900	[0–16,800]
N9a6a	20	8,700	[2,400–14,600]	7,200	[2,700–11,800]	8,700	[0–17,700]
N9a6a1	8	2,500	[300–4,800]	2,600	[200–4,500]	3,000	[0–6,300]
N9a6a4	2	3,900	[0–9,400]	3,900	[0–8,400]	3,900	[0–11,700]
N9a6b	15	200	[0–600]	200	[0–500]	0	[0–0]
N9a8	3	14,600	[8,300–21,200]	14,300	[7,100–21,700]	15,800	[3,200–28,400]
N9a9	3	13,000	[5,300–21,000]	9,700	[3,900–15,700]	10,500	[200–20,800]
N9b	29	17,300	[11,700–23,000]	11,700	[7,500–16,100]	10,600	[4,500–16,700]
N9b1	10	13,900	[8,000–20,000]	12,300	[7,500–17,100]	10,200	[3,900–16,600]
N9b1a	2	12,500	[6,600–18,600]	19,000	[8,800–29,800]	15,800	[300–31,200]
N9b1b	2	3,100	[0–8,700]	2,600	[0–6,200]	7,900	[0–18,800]
N9b1c	5	9,000	[3,000–15,200]	8,400	[3,800–13,200]	7,900	[0–16,100]
N9b1c1+15061	2	7,300	[2,500–12,300]	14,800	[5,900–24,100]	11,800	[0–25,200]
N9b2	4	15,900	[10,000–21,900]	19,700	[11,400–28,400]	17,700	[6,200–29,300]
N9b2+16309	4	14,200	[8,200–20,500]	17,100	[8,800–25,800]	15,800	[3,200–28,400]
R9	134	51,000	[41,200–61,000]	53,300	[40,400–66,600]	54,100	[33,700–74,600]
R9b	45	38,700	[23,900–54,300]	32,800	[20,400–45,900]	31,400	[13,600–49,200]
R9b1	41	22,600	[12,600–33,200]	23,800	[15,000–33,000]	19,800	[8,700–30,900]
R9b1a	32	18,600	[10,800–26,700]	20,700	[13,200–28,500]	23,900	[9,700–38,100]
R9b1a1	18	11,700	[6,000–17,300]	13,400	[6,600–20,300]	9,600	[3,800–15,500]
R9b1a1a	17	9,300	[4,800–13,900]	10,800	[6,200–15,500]	9,300	[3,200–15,300]
R9b1a1a+16192	8	7,800	[1,000–14,900]	11,300	[4,400–18,500]	8,900	[0–18,500]
R9b1a1a+16234	3	7,000	[2,600–11,500]	7,000	[1,500–12,600]	13,100	[0–26,800]
R9b1a1a+7633	3	4,500	[0–15,100]	4,300	[0–9,600]	0	[0–0]
R9b1a2	8	15,900	[8,100–24,000]	15,200	[8,300–22,200]	10,800	[1,600–20,100]
R9b1a2b	5	12,200	[200–25,000]	10,000	[3,700–16,700]	12,600	[0–25,700]
R9b1a2a	3	9,400	[3,300–15,600]	10,600	[4,000–17,400]	7,900	[0–19,400]
R9b1a2a+11077	2	6,000	[0–12,200]	6,600	[800–12,500]	0	[0–0]
R9b1a3	6	10,200	[1,000–19,900]	12,400	[3,700–21,600]	18,400	[1,300–35,500]
R9b1a3+152	5	7,100	[2,100–12,300]	9,000	[1,800–16,400]	22,100	[1,600–42,500]
R9b1b	8	6,700	[800–12,900]	6,500	[2,200–11,100]	3,900	[0–10,100]
R9b2	4	5,700	[1,300–10,200]	5,200	[1,600–9,000]	5,900	[0–12,600]
R9c1	45	28,500	[17,200–40,300]	25,200	[13,200–37,900]	33,100	[9,600–56,600]
R9c1+12618	10	17,700	[7,500–28,500]	13,900	[6,900–21,200]	8,700	[0–17,500]
R9c1+150	2	11,900	[1,500–22,900]	10,600	[3,200–18,400]	11,800	[0–25,200]
R9c1a	33	5,900	[3,700–8,300]	5,100	[2,600–7,800]	6,900	[400–13,500]
R9c1a+11197	10	2,000	[300–3,600]	1,600	[100–3,100]	3,000	[0–7,000]
R9c1a+12123	2	4,00	[800–7,600]	5,200	[100–10,500]	0	[0–0]

R9c1a+1462	5	3,00	[0–7,900]	3,600	[0–8,200]	4,700	[0–14,000]
Y	98	28,00	[16,100–40,500]	24,200	[14,000–34,900]	31,200	[11,600–50,900]
Y1	48	15,500	[8,000–23,300]	10,700	[3,800–17,900]	13,800	[0–27,900]
Y1a	42	4,400	[2,100–6,700]	5,000	[2,500–7,400]	5,100	[1,300–8,800]
Y1a+12397	3	2,300	[0–7,000]	2,600	[0–6,400]	0	[0–0]
Y1a+12732	3	2,900	[700–5,200]	4,000	[1,100–6,800]	2,600	[0–6,300]
Y1a+16266	12	900	[0–2,200]	1,000	[0–2,500]	0	[0–0]
Y1a+16292	8	3,700	[700–6,700]	6,200	[800–11,800]	14,800	[0–29,900]
Y1b	4	10,200	[3,200–17,600]	7,200	[1,600–13,100]	7,900	[0–17,300]
Y1b1	3	3,800	[0–15,100]	1,700	[0–5,100]	5,300	[0–15,600]
Y2	50	9,600	[5,000–14,400]	9,200	[3,100–15,600]	8,800	[0–20,100]
Y2+5435	2	5,000	[0–10,400]	5,200	[100–10,500]	7,900	[0–18,800]
Y2a	43	6,100	[3,200–9,100]	6,700	[2,000–11,500]	8,400	[0–21,400]
Y2a1	36	4,100	[2,300–5,900]	4,500	[2,400–6,600]	1,500	[400–2,700]
Y2a1+146	4	3,000	[0–6,200]	4,600	[0–9,300]	0	[0–0]
Y2a1+228	5	3,300	[1,400–5,200]	4,700	[1,600–7,900]	4,700	[0–10,100]
Y2a1a	7	2,300	[200–4,400]	3,000	[0–6,900]	1,100	[0–3,300]
Y2a1+4767	3	900	[0–5,800]	0	[0–0]	0	[0–0]
Y2b	3	3,400	[0–9,400]	1,700	[0–4,100]	2,600	[0–7,800]
D	176	41,700	[29,500–54,300]	49,900	[31,900–68,900]	43,300	[18,100–68,600]
D5	174	33,300	[24,600–42,200]	34,500	[23,000–46,500]	35,600	[15,500–55,800]
D5a	95	21,000	[13,900–28,500]	20,300	[9,700–31,500]	12,000	[3,600–20,500]
D5a2	88	16,900	[9,100–25,100]	14,900	[6,700–23,500]	11,900	[2,900–21,000]
D5a2+16172	2	14,500	[4,100–25,400]	17,600	[7,800–27,900]	19,700	[2,400–37,000]
D5a2a	83	14,900	[3,300–27,200]	12,100	[5,300–19,200]	11,500	[1,900–21,100]
D5a2a+16092	82	13,600	[8,700–18,800]	9,500	[5,100–13,900]	11,600	[1,900–21,300]
D5a2a1	32	12,800	[8,100–17,600]	14,900	[9,500–20,400]	14,800	[8,100–21,500]
D5a2a1+16092	6	10,800	[6,000–15,700]	11,100	[6,600–15,600]	11,800	[4,100–19,600]
D5a2a1+16172	24	12,000	[8,600–15,700]	13,100	[8,800–17,600]	15,400	[6,800–24,100]
D5a2a1a	10	9,000	[3,600–14,800]	8,400	[4,100–12,900]	12,600	[2,800–22,400]
D5a2a1a1	3	6,600	[1,100–12,400]	6,100	[400–12,000]	5,300	[0–12,500]
D5a2a1a2	5	4,100	[300–8,100]	6,300	[1,700–11,000]	12,600	[1,900–23,300]
D5a2a1b	5	9,500	[3,700–15,500]	11,700	[6,000–17,500]	6,300	[100–12,500]
D5a2a1b+T16092C	2	9,500	[3,100–16,100]	9,700	[3,900–15,700]	7,900	[0–16,800]
D5a2a2	49	1,200	[400–2,000]	1,800	[700–2,900]	1,700	[0–4,100]
D5a2a2+13584	2	800	[0–1,800]	2,600	[0–6,200]	0	[0–0]
D5a2a2+4880	7	600	[0–2,000]	2,600	[0–6,300]	0	[0–0]
D5a3	6	16,900	[9,800–24,200]	15,700	[6,500–25,400]	15,800	[300–31,200]
D5a3a	5	13,400	[6,200–20,900]	13,400	[4,200–23,000]	17,300	[0–35,600]

D5a3a1a	4	3,700	[0–8,600]	3,900	[300–7,600]	2,000	[0–5,800]
D5a3a1a+146	2	1,300	[0–11,800]	1,300	[0–3,800]	0	[0–0]
D5a'b	162	30,600	[22,100–39,400]	30,500	[19,800–41,700]	28,800	[13,600–44,000]
D5b	67	19,300	[13,100–25,500]	17,800	[10,800–25,000]	25,700	[9,700–41,600]
D5b+263	4	8,600	[100–17,400]	12,000	[5,200–19,000]	3,900	[0–9,400]
D5b+263+9992	3	8,600	[3,200–14,100]	11,500	[4,700–18,600]	5,300	[0–12,000]
D5b1	23	17,100	[11,700–22,600]	22,600	[12,600–33,200]	26,800	[9,800–43,700]
D5b1a1	2	13,200	[5,800–20,900]	10,600	[3,200–18,400]	7,900	[0–18,800]
D5b1b	3	12,000	[5,700–18,600]	9,700	[3,900–15,700]	13,200	[1,600–24,700]
D5b1c	16	15,500	[9,900–21,200]	23,200	[10,100–37,000]	29,100	[5,200–52,900]
D5b1c1	15	9,100	[4,000–14,400]	12,300	[4,300–20,600]	21,000	[900–41,100]
D5b1c1+15724	14	6,600	[1,700–11,700]	10,000	[3,400–16,900]	14,500	[0–29,600]
D5b1c1a	11	6,000	[0–13,800]	7,400	[1,200–13,900]	8,600	[0–19,500]
D5b1d	2	9,400	[3,300–15,800]	9,200	[2,300–16,500]	19,700	[2,400–37,000]
D5b3	40	10,900	[5,600–16,400]	9,900	[2,400–17,700]	17,700	[0–37,100]
D5b3+7241	35	3,600	[1,100–6,100]	4,600	[500–8,800]	2,900	[0–6,300]
D5c	12	24,000	[16,000–32,200]	29,000	[16,700–41,900]	15,800	[3,200–28,400]
D5c+16311	3	22,500	[15,200–29,900]	26,800	[16,600–37,400]	28,900	[11,800–46,000]
D5c1+16190	9	13,600	[6,300–21,200]	14,300	[5,400–23,700]	3,500	[100–6,900]

Table S4. Entrance age estimates of the mtDNA lineages in this study in ISEA or Taiwan.

mtDNA lineages	Region	Age estimate (years)	95% confidence interval
N9a6a	ISEA	8,600	2,200 – 15,200
Y2a	ISEA	5,300	2,800 – 7,900
B4b1a2	ISEA	5,300	3,200 – 7,400
	Taiwan	6,700	4,300 – 9,200
B4c1b2a2	ISEA	7,600	4,900 – 10,400
	Taiwan	1,000	300 – 1,800
B5b1c	ISEA (Philippines)	8,900	6,500 – 11,300
R9b1a1a	ISEA	7,900	2,100 – 13,900
R9c1a	ISEA	5,900	3,300 – 8,600
	Taiwan	4,800	1,100 – 8,700
F1a4a1	ISEA	3,400	1,200 – 5,600
F3b1	Taiwan	5,800	700 – 11,100
D5b1c1a	ISEA	4,600	0 – 9,300

Table S5. Founder ages estimates for the main clades in Taiwan.

Founder clade	Age (years)	95% confidence interval
A5b1	0	–
B4a1a	5,900	2,500 –9,300
B4a2	10,100	5,300–15,000
B4b1a2	8,000	4,100–12,100
B5a2	14,100	6,500–22,000
C7a	0	–
D4a	0	–
D4i	0	–
D5b3	10,200	3,800–16,700
D6a2	0	–
E1a	7,100	3,600–10,600
E2b	4,200	1,400–7,100
F1a1d	4,600	300–8,900
F1a3	0	–
F1a3a	9,500	3,700–15,400
F1a4	3,500	0–7,100
F2	0	–
F3b1a	6,600	1,300–12,100
F4b	3,900	400–7,600
M7b1d3	5,600	1,100–10,300
M7b3a	7,700	4,500–10,900
M7c3a	5,700	2,400–9,100
M7c3c	7,400	4,500–10,400
M8a2	0	–
N9a10	8,300	2,300–14,600
R9b1a2	10,100	3,700–16,700
R9c1a	4,800	1,100–8,700
Y2a1a	4,900	1,800–8,000

Supplementary Note 1

Phylogeography of the mtDNA haplogroups targeted in this study

Here we provide, separately, the results of the phylogeographic reconstruction of all mtDNA haplogroups targeted in our study, as well the population expansion signals associated to each mitochondrial DNA lineage.

Haplogroup N9a

The overall phylogeographic pattern of mtDNA haplogroup N9 suggests an Eastern Asian origin ~50 ka (50.6 [37.1; 64.6] ka) (Table 1). N9 encompasses three basal branches: N9a, N9b and Y. The major subclade, N9a, dates to ~20 ka (20.0 [14.5; 25.5] ka) and is frequent across China, Japan and West ISEA (Fig. S1), suggesting an East Asian ancestry of this clade around the time of the LGM. N9a splits into six subclades, four with a strong Northeast Asian (i.e. Chinese and/or Japanese) focus (N9a1'3, N9a2'4'5, N9a8 and N9a9), and two with a probable MSEA/South Chinese ancestry (N9a6 and N9a10).

N9a6, dating to ~15 ka (14.8 [9.9; 19.8] ka), is by far the most frequent subclade of N9 in SEA. This subclade encompasses several basal branches with a strong MSEA ancestry, centred on Vietnam, and two major subclades, N9a6a and N9a6b, present in Austronesian-speaking populations. N9a6a, as shown previously (Hill et al. 2007), is frequent in Malaysia (N9a6a1) and the islands of Borneo and Sumatra (N9a6a2, N9a6a3 and N9a6a4) and, considering its estimated time of entrance in ISEA (8.6 [2.2; 15.2] ka) (Table S4), it probably expanded during the final flooding period (Pelejero et al. 1999).

In contrast with this pattern, subclade N9a10 arose within the region of South China and MSEA, in the same time frame (16.6 [11.0; 22.4] ka), but its major subclade, N9a10a, is mostly present in Taiwan and South China and absent in ISEA (apart from one individual so far in the Batanes Island, most probably related to Taiwan considering the close relationships between the Ivatan and the Yami people (Loo et al. 2011)). Considering the dispersal period of ~10-6 ka (10.0 [4.7; 15.4]; 6.3 [0.2; 12.5] ka) (Table 1) in the region, it seems plausible that N9a10 arrived in Taiwan from China with Neolithic rice agriculturalists, together with M7b1d3, M7b3a and M7c3c (Soares et al. 2015)– but, similar to M7b1d3, there is no sign that it dispersed towards ISEA.

The BSP obtained for mtDNA haplogroup N9a shows two waves of population expansion (Fig. S2), at ~12 ka and ~7 ka (Table 2), the latter one correlating well with the expansion into ISEA and Malaysia, as given by the age of N9a6a. Given the overall phylogenetic and phylogeographic pattern and the BSP population expansions, mtDNA haplogroup N9a appears to have an East Asian origin with some of its lineages spreading to the Sunda continent during the postglacial period, thus constituting a strong candidate for a postglacial mtDNA signal for migrations from MSEA.

Haplogroup Y

A second branch of N9, haplogroup Y, dates to almost 30 ka (28.0 [16.1; 40.5] ka) (Table 1). This splits into two major subclades, Y1, which shows a clear North Asian ancestry, probably within South Siberia at the end of Last Glacial period, and a second younger subclade Y2, dating to ~10 ka (9.6 [5.0; 14.4] ka), which is mostly found in Taiwan and ISEA, at an overall frequency of ~18%. Y2a, dating to ~6 ka (6.1 [3.2; 9.1] ka), is the most frequent branch of Y2, and the only one observed in Austronesian-speaking populations. The age interval between Y2 (9.6 ka) and Y2a (6 ka) suggests a migration from mainland to insular locations in the time frame of the hypothetical settlement of Taiwan by rice-agriculturalists from South China. Y2a is frequent in Taiwan and ISEA, where its founder age suggested an entrance in ISEA around ~5 ka (5.3 [2.8; 7.9] ka). Y2a encompasses a "star-like" branch, Y2a1a that is mainly found in ISEA which, given its age (4.1 [2.3; 5.8] ka), seems likely to have accompanied the Neolithic Austronesian dispersal from Taiwan into ISEA. The BSP also displays a population increment after 5 ka. Y2, mostly Y2a1, thereby represents, along with M7c3c (Soares et al. 2015), a strong candidate for an OOT mtDNA marker.

Haplogroup B4b1

Haplogroup B4 (Hill et al. 2007) is subdivided into three main subclades, B4a, B4b'd and B4c, and dates to ~35 ka (Derenko et al. 2012). Subclade B4b splits in two major subclades, the Amerindian-specific branch B2 (Bandelt et al. 2003; Eshleman et al. 2003) and B4b1, dating to ~25 ka (25.1 [17.0; 33.6] ka) (Table 1), which displays a wide geographic distribution from Northeast Asia to SEA (Fig. S1).

We previously identified B4b1 as a candidate genetic marker of the pottery-making rice-farming OOT dispersal into ISEA (Soares et al. 2015). The reconstructed whole-mtDNA genome phylogeography shows that within B4b1, B4b1a is by far the most frequent subclade in SEA, found in both MSEA and ISEA. This subclade is also found in Northern Asia, in the form of B4b1a1 and B4b1a3.

B4b1a2, dating to ~9 ka (9.3 [6.8; 11.8] ka), is the major subclade of B4b1a, and is the only one that is present in Taiwan and ISEA. A founder age from ISEA into Taiwan indicates an entrance ~7 ka (6.7 [4.3; 9.1] ka), matching the hypothetical rice-agriculturalist migration from China to Taiwan. Given the overall phylogeographic patterns of this clade, with a founder age of ~5 ka (5.3 [3.2; 7.4] ka) in ISEA, it seems possible that B4b1a2 could have dispersed from South China into Taiwan and later to ISEA in a similar fashion to M7c3c, following the OOT model. Another similarity with M7c3c is that the clade expanded to Micronesia and northwest Polynesia and is not detected along the North Coast of New Guinea or the Bismarck Archipelago. The increment time shown by the BSP (Table 2) within the Neolithic time frame also supports B4b1a2 as a genetic signal for the Neolithic dispersal from Taiwan to ISEA.

Haplogroup B4c1

mtDNA haplogroup B4c has a pre-LGM Northern Asian origin, followed by a later distribution of its major subclade B4c1 and minor subclade B4c2 (Derenko et al. 2012). B4c1 is broadly frequent throughout East Asia and SEA (Fig. S1). It splits into two further subclades: a minor Japanese offshoot, B4c1c, and a second major subclade, B4c1a'b, incorporating B4c1a, displaying a clear Northeast Asian ancestry centred on Japan, and B4c1b which, by contrast, is prominent throughout Malays, Filipinos and aboriginal Taiwanese. Within B4c1b, the subclade B4c1b2 – more specifically the branch B4c1b2a2, dating to ~8 ka (8.0[5.6; 10.5] ka) (Table 1) – is by far the most frequent lineage in Taiwan and ISEA, suggesting an ancestry within those regions.

Soares et al. (2015), based on mtDNA HVS-I data, suggested that B4c1 could represent a genetic marker for the OOT model. At the whole-mtDNA genome level, only one subclade of B4c1b2a2 is found in Taiwan, whereas several exist in ISEA, including the branch that appears in Taiwan (detected in the Philippines). This evidence points to an origin in ISEA and a northwards migration. Using an exploratory founder analysis, considering ISEA and Taiwan as hypothetical source and sink populations and *vice versa*, we estimated a founder age of ~1 ka (1.0 [0.3; 1.7] ka) in Taiwan and 7.6 [4.9; 10.4] ka in ISEA, emphasizing that an origin in Taiwan is very unlikely. The increment ~6 ka in the BSP (Table 2), as for B4b1 above, does not clearly distinguish between an OOT or a postglacial expansion. The age of B4c1b2a at 14.5 [6.4; 23.1] ka indicates that the clade entered ISEA between 15 ka and 8 ka, a pattern similar to the more common B4a1a1 (Soares et al. 2011).

In fact, the B4c1b2a2a subclade (defined here) is the only Taiwanese branch of B4c1, and is found only in the Yami. These people, also known as Tao, are native to the small outlying Orchid Island in Taiwan and are distinct from other Taiwanese aboriginal groups as the only non-Formosan Austronesian speakers among Taiwanese aborigines. The languages of Yami belong to the Batanic sub-branch of the Western Malayo-Polynesian branch, which comprises all of the Austronesian languages spoken outside Taiwan(Blust 2009), suggesting a recent migration from the Philippines. This hypothesis is supported by the recent founder age ~1 ka as calculated above. Given the overall pattern, B4c1b2a2 appears to constitute a genetic signature of the reticulated network of cultural/linguistic relationships between Orchid Island and Philippines previously described by Ross (Ross 2005). Considering that B4c1b2a2 is not defined by any HVS-I variants, the diversity detected outside ISEA was probably part of B4c1b2a* which allowed the clade to be considered a founder into ISEA in the HVS-I founder analysis (Soares et al. 2015) under the founder analysis criteria for derived clades in the source (Richards et al. 2000). This reinforces the need to study phylogeographic patterns at the whole-mtDNA level for optimal results.

Haplogroup B5b

The mtDNA haplogroup B5b is the other Asian haplogroup B lineage that we are focusing on in our study. B5b reaches up to ~8–9% in Malaysia and southeast Indonesia, but it is considerably less frequent in the neighbouring regions (Fig. S1). B5b dates to ~30 ka (29.8 [20.7; 39.3] ka) (Table 1), and the major subclade, B5b1, diverged ~24 ka (23.9 [13.9; 34.3] ka) somewhere within East Asia. B5b1 splits into two main subclades: B5b1a is an entirely Japanese clade that dates to ~19 ka (19.0 [8.8; 29.8] ka) and B5b1c

is found only in Austronesian-speaking populations, with a founder estimate age in the Philippines ~9 ka (8.8 [6.5; 11.3] ka). The time gap between this Holocene subclade and its Late Pleistocene ancestor (B5b1) suggests extensive genetic drift during this period, most likely due to the sea-level rises that resulted from the global warming at the end of the Pleistocene (Pelejero et al. 1999). The BSPs also show two-stepped population growth in the Late Pleistocene and Holocene (Table 2).

Given the overall pattern, B5b seems to have had a South Chinese origin in the Late Pleistocene and to have spread widely over central/eastern Asia since then, similar to B4c1b2a2 (above) and B4a1a (Soares et al. 2011),and its arrival in ISEA was prompted by climate change, rather than driven by the Neolithic (from MSEA in this case, not Taiwan) as inferred from the HVS-I founder analysis performed previously (Soares et al. 2015). Again, the fact that B5b1c (or even B5b1) is not defined by any HVS-I mutation led the clade as a whole to be considered a founder from Asia, resulting in very imprecise age estimates.

Haplogroup R9b

mtDNA haplogroup R9 encompasses three basal branches, R9b, R9c and F, all with an East Asian origin in the Late Pleistocene (Hill et al. 2006; Peng et al. 2010). R9b is frequent throughout East Asia, reaching ~20% in South China. The presence of this clade gradually decreases throughout continental and insular Southeast Asia, and it is completely absent in the Philippines (Fig. S1). R9b was identified as a possible marker for postglacial expansions by Hill et al. (2006). Here we reanalysed the R9b phylogeography in light of the whole-mtDNA sequences, as well as our re-evaluation of the molecular clock (Soares et al. 2009).

R9b dates to ~39 ka (38.7 [23.9; 54.3]) ka (Table 1), and splits into two main branches: R9b1, with a clear, ancient MSEA ancestry, and a more recent subclade, R9b2, dating to just ~6 ka (5.7 [1.3; 10.2] ka), only found in Malays, Vietnamese and Thai. This implies an overall origin of R9b in MSEA. R9b1a, the major clade of R9b, dating to ~18 ka (18.6 [10.8; 26.7] ka), splits into three subclades, R9b1a1, R9b1a2 and R9b1a3. The phylogeographic distribution of R9b1a1 suggests an expansion within the SEA ~11 ka (11.6 [6.0; 17.3] ka), with its subclade, R9b1a1a, showing a postglacial founder estimate age in insular Southeast Asia of ~8 ka (7.9 [2.1; 13.9] ka).

This overall pattern, allied to the population expansion detected in the Mid-Holocene (Fig. S2), supports the view of R9b as a genetic marker for postglacial expansions from MSEA, as suggested before (Hill et al. 2006). The mtDNA haplogroup R9b – similarly to N9a – has an East Asian origin in the Late Pleistocene, and later with the climatic improvement dispersed to MSEA and southwards towards Malaysia and ISEA at the time of sea-level rises.

Haplogroup R9c

R9c, which is mostly found on the eastern side of ISEA (Fig. S1), has only one major subclade, R9c1, dating to ~28 ka (28.4 [17.2-40.3] ka) (Table 1). This subclade splits into three main branches, all of them

with a tree structure and distribution focused on ISEA. The main branch R9c1a, dating to ~6 ka (5.9 [3.7; 8.3] ka), is largely restricted to Austronesian-speaking populations, excepting one South Chinese sample. Following previous inferences (Soares et al. 2015), R9c1a appears to have entered ISEA at the end of the postglacial dispersals (~5.9 [3.3; 8.6] ka), later reaching Taiwan (~4.8 [1.1; 8.7] ka). This was mainly inferred from a distribution centred on the Sunda shelf and the complete lack of HVS-I diversity in Taiwan. However at the whole-mtDNA level the picture becomes less clear-cut. There is a single South Chinese sample that could represent an accidental or indicate an origin in South China/Taiwan. Also, the age estimate of the clades at ~6 ka is ambiguous and slightly older than the hypothetical OOT migration, but it is also more recent than the postglacial expansions. The large age distance between R9c1a and its ancestor, R9c1 (nearly 30 ka (28.5 [17.2-40.3] ka)) indicate that this subclade could have been within ISEA or Taiwan since the Late Pleistocene, a pattern mainly observed in clades present in ISEA that went through extensive genetic drift during the flood episodes (as B4a1a1, E, B4c1b2a2, and B5b1). The recent population expansion (~2 to 5 ka) (Table 2) detected by the BSP could indicate a more recent autochthonous Southeast Asian expansion.

Overall, R9c does not fit the OOT phylogeographic parameters established by Soares et al. (2015) and shows ambiguous results; but the overall pattern suggests an ISEA origin rather than an OOT origin.

Haplogroup F1a4

The mtDNA haplogroup F1a4 is extremely rare, found at low frequency only in South China and Austronesian-speaking populations. F1a4 dates to 16.2 [7.0; 26.0] ka (Table 1) and includes a major subclade, F1a4a, with a clear Chinese origin ~12 ka (11.7 [3.0; 20.9] ka) (Table 1). This clade encompasses a star-like cluster of several Taiwanese ancestors, referred as F1a4a1, dating to just above 4 ka (4.3 [1.8; 6.8] ka), and a descendent subclade, F1a4a1a, found only in ISEA, Malaysia and Micronesia, dating just over 3 ka (3.3 [1.3; 5.3] ka). The time gap between the emergence of F1a4a and this F1a4a1 suggests a time of arrival in Taiwan fitting well the time frame for the arrival of rice-agriculturalists in the OOT model. An entry into ISEA between the age of F1a4a1 in Taiwan (4.3 ka) and F1a4a1a at 3.3 ka also fits the Austronesian migration in the OOT model. The BSP plot of F1a4 shows population increment within the last 4 ka (Table 2), which fits the arrival/dispersal time of F1a4a1 in ISEA (~3.4 [1.2; 5.6] ka). Given this overall pattern, F1a4 could have been carried into Taiwan from South China by rice-agriculturists, and later with the OOT migration into ISEA along with mtDNA lineages B4b1a2, Y2a1 and M7c3c (Soares et al. 2015). As with M7c3c and B4b1a2, the presence of this clade in the Pacific is evident only in Micronesia (suggesting an arrival there directly from ISEA).

Haplogroup F3

The mtDNA haplogroup F3 dates to ~32 ka (31.7 [21.5; 42.3] ka) (Table 1) and is fairly common throughout East and Southeast Asia. There are two major basal subclades, F3a and F3b, both with similar Late Pleistocene ages (26.7 [16.5; 37.1] ka and 25.2 [15.4; 35.4] ka, respectively) (Table 1), but with

clearly different ancestries. F3a is mostly present in MSEA, such as Vietnam, Laos, Malaysia and southern China, suggesting that this clade has a MSEA ancestry. The daughter clade, F3a1, dates to ~16 ka (16.6 [9.0; 24.5] ka) and, similarly to its ancestral clade, displays a MSEA origin centred on Vietnam and Laos.

The sister clade F3b is divided into two subclades. One, F3b2, is rare and was detected only in South China, while F3b1, dating to ~12 ka (12.4 [5.2; 20.0] ka), is by far the more common subclade of F3b and is largely restricted to Austronesian-speaking populations in ISEA and Taiwan. Within F3b1, F3b1b is restricted to ISEA while F3b1a is found in ISEA and Taiwan, strongly suggesting an origin in ISEA and a migration into Taiwan. A founder age into Taiwan (5.8 [0.7; 11.1] ka) (Table S4), is concordant with the hypothesis that this clade accompanied postglacial dispersed from ISEA towards Taiwan, again most probably as a result of sea-level rises. Overall, indeed, the age and distribution of haplogroup F3 shows many similarities with haplogroup E (Soares et al. 2008). It likewise emerged in ancient Sundaland over 30 ka, but probably further to the west, within what is now MSEA. Two subclades within haplogroup F3b1 show traces of expansion in the last 8 ka in ISEA, with one reaching Taiwan. The BSP for mtDNA haplogroup F3 shows two population expansion periods, the first between ~5–10 ka and the second within the last 4 ka (Table 2). Given the phylogeographic and phylogenetic patterns of F3 overall, it seems likely that this clade dispersed more than once within the Sunda region over the last ~16 ka.

Haplogroup D5

The mtDNA haplogroup D5 dates to just over ~30 ka (33.3 [24.6; 42.2] ka), and is widely distributed throughout East and Southeast Asia. There are two basal branches, D5a’b and D5c. The latter further separates into D5c1 and D5c2, both with a probable North/Northeast Asian origin. D5a’b separates into two major subclades: D5a, which is widely dispersed throughout East and Northeast Asia, and D5b, which is extremely frequent in Taiwan and less frequent in Southeast Asia. This clade splits into two subclades, D5b1 and D5b3 (a newly defined branch). D5b3 dates to ~11 ka (10.9 [5.6; 16.4] ka) (Table 1) and is largely restricted to Chinese and Taiwanese populations, and virtually absent in ISEA. Given the existence of several Taiwanese branches dating to less than 4 ka, it seems likely that D5b3 moved between 10 ka and 3 ka, suggesting that it could have arrived in Taiwan with the Neolithic rice-farmers from South China. However, it did not follow the Austronesian movement OOT, resembling in thsi respect the patterns of mtDNA haplogroups N9a10a and M7b1d3.

Within D5b1, subclade D5b1c1, dating to ~9 ka (9.1 [4.0; 14.4] ka) is the only D5 subclade to disperse to insular Southeast Asia. This subclade includes a cluster with ancestry in Taiwan, D5b1c1a, dating ~6 ka (6.0 [0; 13.8] ka), restricted to Austronesian-speaking populations. Although the tree might seem to imply a deeper ancestry in ISEA than in Taiwan, this is caused by a single HVS-I variant, 16092, that is mildly fast and could represent homoplasy. A founder age into ISEA is ~4.6 [0; 9.3] ka, again suggesting a Neolithic OOT marker clade. The population increase between ~13 ka till ~3.5 ka, with a peak at ~7.7 ka (Table 2), mostly shows a signal of early population expansion within South China. In contrast to other clades described above, the Austronesian component in the BSP is somewhat low which does not make

any hypothetical OOT expansion important in the overall BSP against postglacial expansions in continental Asia. Although it is a probable OOT marker its presence in ISEA is low, at comparable levels to another OOT candidate, M7b3 (Soares et al. 2015).

References

Bandelt H-J, Achilli A, Kong Q-P, Salas A, Lutz-Bonengel S, Sun C, Zhang Y-P, Torroni A, Yao Y-G (2005) Low “penetrance” of phylogenetic knowledge in mitochondrial disease studies. Biochem Biophys Res Commun 333:122-130.

Bandelt H-J, Herrnstadt C, Yao YG, Kong QP, Kivisild T, Rengo C, Scozzari R, Richards M, Villems R, Macaulay V, Howell N, Torroni A, Zhang YP (2003) Identification of Native American founder mtDNAs through the analysis of complete mtDNA sequences: some caveats. Ann Hum Genet 67:512-524.

Behar DM, van Oven M, Rosset S, Metspalu M, Loogvali EL, Silva NM, Kivisild T, Torroni A, Villems R (2012) A "Copernican" reassessment of the human mitochondrial DNA tree from its root. Am J Hum Genet 90:675-684.

Bi R, Zhang A-M, Jia X, Zhang Q, Yao Y-G (2012) Complete mitochondrial DNA genome sequence variation of Chinese families with mutation m. 3635G> A and Leber hereditary optic neuropathy. Mol Vis 18:3087.

Bilal E, Rabadan R, Alexe G, Fuku N, Ueno H, Nishigaki Y, Fujita Y, Ito M, Arai Y, Hirose N (2008) Mitochondrial DNA haplogroup D4a is a marker for extreme longevity in Japan. PLoS One 3:e2421.

Blust R (2009) The Austronesian languages. Pacific Linguistics, Canberra, Australia

Delfin F, Ko AM-S, Li M, Gunnarsdóttir ED, Tabbada KA, Salvador JM, Calacal GC, Sagum MS, Datar FA, Padilla SG (2014) Complete mtDNA genomes of Filipino ethnolinguistic groups: a melting pot of recent and ancient lineages in the Asia-Pacific region. European Journal of Human Genetics : EJHG 22:228-237.

Derenko M, Malyarchuk B, Denisova G, Perkova M, Rogalla U, Grzybowski T, Khusnutdinova E, Dambueva I, Zakharov I (2012) Complete mitochondrial DNA analysis of eastern Eurasian haplogroups rarely found in populations of northern Asia and eastern Europe. PLoS One 7:e32179.

Derenko M, Malyarchuk B, Grzybowski T, Denisova G, Dambueva I, Perkova M, Dorzhu C, Luzina F, Lee HK, Vanecek T (2007) Phylogeographic analysis of mitochondrial DNA in northern Asian populations. Am J Hum Genet 81:1025-1041.

Derenko M, Malyarchuk B, Grzybowski T, Denisova G, Rogalla U, Perkova M, Dambueva I, Zakharov I (2010) Origin and post-glacial dispersal of mitochondrial DNA haplogroups C and D in northern Asia. PloS One 5:e15214.

Duggan AT, Whitten M, Wiebe V, Crawford M, Butthof A, Spitsyn V, Makarov S, Novgorodov I, Osakovsky V, Pakendorf B (2013) Investigating the prehistory of Tungusic Peoples of Siberia and the Amur-Ussuri Region with complete mtDNA genome sequences and Y-chromosomal markers. PloS One 8:e83570.

Eshleman JA, Malhi RS, Smith DG (2003) Mitochondrial DNA studies of Native Americans: conceptions and misconceptions of the population prehistory of the Americas. Evol Anthropol 12:7-18.

Gunnarsdóttir ED, Li M, Bauchet M, Finstermeier K, Stoneking M (2011a) High-throughput sequencing of complete human mtDNA genomes from the Philippines. Genome Res 21:1-11.

Gunnarsdóttir ED, Nandineni MR, Li M, Myles S, Gil D, Pakendorf B, Stoneking M (2011b) Larger mitochondrial DNA than Y-chromosome differences between matrilocal and patrilocal groups from Sumatra. Nat Commun 2:228.

Hartmann A, Thieme M, Nanduri LK, Stempfl T, Moehle C, Kivisild T, Oefner PJ (2009) Validation of microarray-based resequencing of 93 worldwide mitochondrial genomes. Hum Mutat 30:115-122.

Hill C, Soares P, Mormina M, Macaulay V, Clarke D, Blumbach PB, Vizuete-Forster M, Forster P, Bulbeck D, Oppenheimer S, Richards M (2007) A mitochondrial stratigraphy for Island Southeast Asia. Am J Hum Genet 80:29-43.

Hill C, Soares P, Mormina M, Macaulay V, Meehan W, Blackburn J, Clarke D, Raja JM, Ismail P, Bulbeck D, Oppenheimer S, Richards M (2006) Phylogeography and ethnogenesis of aboriginal Southeast Asians. Mol Biol Evol 23:2480-2491.

Ingman M, Gyllensten U (2007) Rate variation between mitochondrial domains and adaptive evolution in humans. Hum Mol Genet 16:2281-2287.

Ji F, Sharpley MS, Derbeneva O, Alves LS, Qian P, Wang Y, Chalkia D, Lvova M, Xu J, Yao W (2012) Mitochondrial DNA variant associated with Leber hereditary optic neuropathy and high-altitude Tibetans. PNAS 109:7391-7396.

Jinam TA, Hong LC, Phipps ME, Stoneking M, Ameen M, Edo J, Saitou N (2012) Evolutionary history of continental Southeast Asians: "Early train" hypothesis based on genetic analysis of mitochondrial and autosomal DNA data. Mol Biol Evol. 29:3513-3527

Kazuno AA, Munakata K, Mori K, Tanaka M, Nanko S, Kunugi H, Umekage T, Tochigi M, Kohda K, Sasaki T (2005) Mitochondrial DNA sequence analysis of patients with ‘atypical psychosis’. Psychiatry Clin Neurosci 59:497-503.

Ko AM-S, Chen C-Y, Fu Q, Delfin F, Li M, Chiu H-L, Stoneking M, Ko Y-C (2014) Early Austronesians: into and out of Taiwan. Am J Hum Genet 94:426-436.

Kong Q-P, Bandelt H-J, Sun C, Yao Y-G, Salas A, Achilli A, Wang C-Y, Zhong L, Zhu C, Wu S-F, Torroni A, Zhang Y-P (2006) Updating the East Asian mtDNA phylogeny: a prerequisite for the identification of pathogenic mutations. Hum Mol Genet 15:2076 - 2086.

Kong Q-P, Yao Y-G, Sun C, Bandelt H-J, Zhu C-L, Zhang Y-P (2003) Phylogeny of East Asian mitochondrial DNA lineages inferred from complete sequences. Am J Hum Genet 73:671-676.

Kumar S, Ravuri RR, Koneru P, Urade B, Sarkar B, Chandrasekar A, Rao V (2009) Reconstructing Indian-Australian phylogenetic link. BMC Evol Biol 9:173.

Loo J-H, Trejaut JA, Yen J-C, Chen Z-S, Lee C-L, Lin M (2011) Genetic affinities between the Yami tribe people of Orchid Island and the Philippine Islanders of the Batanes archipelago. BMC Genet 12:21.

Macaulay V, Hill C, Achilli A, Rengo C, Clarke D, Meehan W, Blackburn J, Semino O, Scozzari R, Cruciani F, Taha A, Shaari NK, Raja JM, Ismail P, Zainuddin Z, Goodwin W, Bulbeck D, Bandelt H-J, Oppenheimer S, Torroni A, Richards M (2005) Single, rapid coastal settlement of Asia revealed by analysis of complete mitochondrial genomes. Science 308:1034-1036.

Malyarchuk B, Derenko M, Denisova G, Kravtsova O (2010) Mitogenomic diversity in Tatars from the Volga-Ural region of Russia. Mol Biol Evol 27:2220-2226.

Mielnik-Sikorska M, Daca P, Malyarchuk B, Derenko M, Skonieczna K, Perkova M, Dobosz T, Grzybowski T (2013) The history of Slavs inferred from complete mitochondrial genome sequences. PloS One 8:e54360.

Mishmar D, Ruiz-Pesini E, Golik P, Macaulay V, Clark AG, Hosseini S, Brandon M, Easley K, Chen E, Brown MD (2003) Natural selection shaped regional mtDNA variation in humans. PNAS 100:171-176.

Pelejero C, Kienast M, Wang L, Grimalt JO (1999) The flooding of Sundaland during the last deglaciation: Imprints in hemipelagic sediments from the southern South China Sea. Earth and Planetary Science Letters 171:661-671.

Peng M-S, Quang HH, Dang KP, Trieu AV, Wang H-W, Yao Y-G, Kong Q-P, Zhang Y-P (2010) Tracing the Austronesian footprint in Mainland Southeast Asia: a perspective from mitochondrial DNA. Mol Biol Evol 27:2417-2430.

Reiff DM, Spathis R, Chan CW, Vilar MG, Sankaranarayanan K, Lynch D, Ehrlich E, Kerath S, Chowdhury R, Robinowitz L (2011) Inherited and somatic mitochondrial DNA mutations in Guam amyotrophic lateral sclerosis and parkinsonism-dementia. J Neurol Sci 32:883-892.

Richards M, Macaulay V, Hickey E, Vega E, Sykes B, Guida V, Rengo C, Sellitto D, Cruciani F, Kivisild T, Villems R, Thomas M, Rychkov S, Rychkov O, Rychkov Y, Gölge M, Dimitrov D, Hill E, Bradley D, Romano V, Cali F, Vona G, Demaine A, Papiha S, Triantaphyllidis C, Stefanescu G, Hatina J, Belledi M, Di Rienzo A, Novelletto A, Oppenheim A, Nørby S, Al-Zaheri N, Santachiara-Benerecetti S, Scozzari R, Torroni A, Bandelt H-J (2000) Tracing European founder lineages in the Near Eastern mtDNA pool. Am J Hum Genet 67:1251-1276.

Ross M (2005) The Batanic languages in relation to the early history of the Malayo-Polynesian subgroup of Austronesian. Journal of Austronesian Studies 1:1-24.

Scholes C, Siddle K, Ducourneau A, Crivellaro F, Jarve M, Rootsi S, Bellatti M, Tabbada K, Mormina M, Reidla M, Villems R, Kivisild T, Lahr MM, Migliano AB (2011) Genetic diversity and evidence for population admixture in Batak Negritos from Palawan. Am J Phys Anthropol 146:62-72.

Shin WS, Tanaka M, Suzuki J, Hemmi C, Toyo-oka T (2000) A novel homoplasmic mutation in mtDNA with a single evolutionary origin as a risk factor for cardiomyopathy. Am J Hum Genet 67:1617-1620.

Soares P, Ermini L, Thomson N, Mormina M, Rito T, Röhl A, Salas A, Oppenheimer S, Macaulay V, Richards MB (2009) Correcting for purifying selection: an improved human mitochondrial molecular clock. Am J Hum Genet 84:740-759.

Soares P, Rito T, Trejaut J, Mormina M, Hill C, Tinkler-Hundal E, Braid M, Clarke DJ, Loo J-H, Thomson N, Denham T, Donohue M, Macaulay V, Lin M, Oppenheimer S, Richards MB (2011) Ancient voyaging and Polynesian origins. Am J Hum Genet 88:239-247.

Soares P, Trejaut JA, Loo JH, Hill C, Mormina M, Lee CL, Chen YM, Hudjashov G, Forster P, Macaulay V, Bulbeck D, Oppenheimer S, Lin M, Richards MB (2008) Climate change and postglacial human dispersals in Southeast Asia. Mol Biol Evol 25:1209-1218.

Soares P, Trejaut JA, Rito T, Cavadas B, Hill C, Eng KK, Mormina M, Brandão A, Fraser RM, Wang T-Y, Loo J-H, Snell C, Ko T-M, Amorim A, Pala M, Macaulay V, Bulbeck D, Wilson JF, Gusmão L, Pereira L, Oppenheimer S, Lin M, Richards MB (2015) Resolving the ancestry of Austronesian-speaking populations. Hum Genet (in press) doi: 10.1007/s00439-015-1620-z.

Starikovskaya EB, Sukernik RI, Derbeneva OA, Volodko NV, Ruiz-Pesini E, Torroni A, Brown MD, Lott MT, Hosseini SH, Huoponen K (2005) Mitochondrial DNA diversity in indigenous populations of the southern extent of Siberia, and the origins of Native American haplogroups. Ann Hum Genet 69:67-89.

Sukernik R, Volodko N, Mazunin I, Eltsov N, Starikovskaya E (2010) The genetic history of Russian old settlers of polar northeastern Siberia. Russ J Genet 46:1386-1394.

Sukernik RI, Volodko NV, Mazunin IO, Eltsov NP, Dryomov SV, Starikovskaya EB (2012) Mitochondrial genome diversity in the Tubalar, Even, and Ulchi: contribution to prehistory of native Siberians and their affinities to Native Americans. Am J Phys Anthropol 148:123-138.

Summerer M, Horst J, Erhart G, Weißensteiner H, Schönherr S, Pacher D, Forer L, Horst D, Manhart A, Horst B (2014) Large-scale mitochondrial DNA analysis in Southeast Asia reveals evolutionary effects of cultural isolation in the multi-ethnic population of Myanmar. BMC Evol Biol 14:17.

Tabbada KA, Trejaut J, Loo JH, Chen YM, Lin M, Mirazon-Lahr M, Kivisild T, De Ungria MC (2010) Philippine mitochondrial DNA diversity: a populated viaduct between Taiwan and Indonesia? Mol Biol Evol 27:21-31.

Tanaka M, Cabrera VM, González AM, Larruga JM, Takeyasu T, Fuku N, Guo L-J, Hirose R, Fujita Y, Kurata M (2004) Mitochondrial genome variation in eastern Asia and the peopling of Japan. Genome Res 14:1832-1850.

The 1000 Genomes Project Consortium (2012) An integrated map of genetic variation from 1,092 human genomes. Nature 491:56-65.

Ueno H, Nishigaki Y, Kong Q-P, Fuku N, Kojima S, Iwata N, Ozaki N, Tanaka M (2009) Analysis of mitochondrial DNA variants in Japanese patients with schizophrenia. Mitochondrion 9:385-393.

Volodko NV, Starikovskaya EB, Mazunin IO, Eltsov NP, Naidenko PV, Wallace DC, Sukernik RI (2008) Mitochondrial genome diversity in Arctic Siberians, with particular reference to the evolutionary history of Beringia and Pleistocenic peopling of the Americas. Am J Hum Genet 82:1084-1100.

Wang C-Y, Wang H-W, Yao Y-G, Kong Q-P, Zhang Y-P (2007) Somatic mutations of mitochondrial genome in early stage breast cancer. Int J Cancer 121:1253-1256.

Zhang X, Qi X, Yang Z, Serey B, Sovannary T, Bunnath L, Seang Aun H, Samnom H, Zhang H, Lin Q, van Oven M, Shi H, Su B (2013) Analysis of mitochondrial genome diversity identifies new and ancient maternal lineages in Cambodian aborigines. Nat Commun 4:2599.

Zou Y, Jia X, Zhang A-M, Wang W-Z, Li S, Guo X, Kong Q-P, Zhang Q, Yao Y-G (2010) The < i> MT-ND1</i> and< i> MT-ND5</i> genes are mutational hotspots for Chinese families with clinical features of LHON but lacking the three primary mutations. Biochem Biophys Res Commun 399:179-185.
